# Functionalized Electrospun Nanofibers as a Versatile Platform for Colorimetric Detection of Heavy Metal Ions in Water: A Review

**DOI:** 10.3390/ma13102421

**Published:** 2020-05-25

**Authors:** Brabu Balusamy, Anitha Senthamizhan, Tamer Uyar

**Affiliations:** Department of Fiber Science & Apparel Design, College of Human Ecology, Cornell University, Ithaca, NY 14853, USA

**Keywords:** electrospinning, nanofiber, water pollution, heavy metal, colorimetric detection, sensor, direct blending, surface functionalization

## Abstract

The increasing heavy metal pollution in the aquatic ecosystem mainly driven by industrial activities has raised severe concerns over human and environmental health that apparently necessitate the design and development of ideal strategies for the effective monitoring of heavy metals. In this regard, colorimetric detection provides excellent opportunities for the easy monitoring of heavy metal ions, and especially, corresponding solid-state sensors enable potential opportunities for their applicability in real-world monitoring. As a result of the significant interest originating from their simplicity, exceptional characteristics, and applicability, the electrospun nanofiber-based colorimetric detection of heavy metal ions has undergone radical developments in the recent decade. This review illustrates the range of various approaches and functional molecules employed in the fabrication of electrospun nanofibers intended for the colorimetric detection of various metal ions in water. We highlight relevant investigations on the fabrication of functionalized electrospun nanofibers encompassing different approaches and functional molecules along with their sensing performance. Furthermore, we discuss upcoming prospectus and future opportunities in the exploration of designing electrospun nanofiber-based colorimetric sensors for real-world applications.

## 1. Introduction

Water is the resource of human life that is more necessary than any other, and upholding its safety and quality is a great challenge in the 21st century. The water quality has been significantly endangered by the occurrence of various chemical and biological pollutants that pose a severe threat to the aquatic environment and human health. The recent report of the World Health Organization (WHO) states that still billions of people around the globe lack access to safely managed water and thus, it is important to continue to establish developmental goals to achieve water quality and sanitation [1]. Although chemicals have improved the quality of human life, their continuous development and corresponding environmental pollution leads to detrimental effects through a variety of exposure pathways. Still, the determination of their persistence, transformation, and accumulation behavior in different environmental compartments remains a challenge for defining their complete associated risks, since not all the chemicals are equivocal nor similar in exposure [2,3]. Moreover, the aquatic environment encompassed a variety of molecules that drive continuous interaction with chemical pollutants and the mixtures are expected to cause severe effects greater than their own individual threshold limits resulting from the “concentration addition and independent action” phenomenon of the chemicals with similar and different modes of action, respectively [4,5].

Heavy metals are relatively dense elements with high atomic weight that are excessively soluble, non-degradable, and readily absorbed nature by different biota in the aquatic environment; they are also considered as the most dominant chemical pollutant of the water resources and expected to persist in the environment from decades to centuries, resulting in an extreme risk of easy introduction into the food chain and biomagnification. Although heavy metals are naturally occurring in the environment, the discharge of effluents containing heavy metals from several industries is the primary source of water pollution [6,7,8,9]. The heavy metals possess deleterious health risks even at very lesser concentration with a wide spectrum of potential harmful effects on neurological, cardiovascular, gastrointestinal, musculoskeletal, endocrine, renal, hepatic, reproductive, and developmental systems through various mechanistic actions [10,11,12,13]. Upon exposure through various routes, the heavy metals tend to disrupt the metabolic process of the human body through affecting the proteins, enzymatic activities, glycolysis, and producing reactive oxygen species, thus resulting in the interruption of cellular signaling pathways, calcium signaling pathways, lipid peroxidation, and oxidative DNA damage, which obviously leads to cause damage and cancer in the vital organs [14,15,16,17,18,19]. Therefore, the United States Environmental Protection Agency (US EPA) and the WHO have set standard values for the maximum allowable concentration of toxic metals in water intended for human consumption to ensure the quality of water [20,21]. Furthermore, few most hazardous heavy metals are even restricted by the European Union’s Restriction on Hazardous Substances (RoHS) directive for their use in electronic and electrical equipment [22]. Therefore, monitoring these contaminants in the water resources is a primary concern for protecting human health against the negative impacts attributed to heavy metal toxicity.

Indeed, improving awareness has inspired several research efforts over the decades for monitoring heavy metals in the water for upholding its quality and safety. Despite several sophisticated analytical instrumentation techniques available for the detection of metal ions in the water [23,24], the rise of nanotechnology has indubitably enabled the field to take a huge step forward. It is fair to state from the key findings that enormous nanomaterials have witnessed breakthrough contributions owing to their excellent size, structural, and tailoring properties for enhanced analyte detection by employing several approaches, including electrochemical, biological, and optical sensing. Note that successful nanomaterials exploited for metal ions detection using various approaches include carbon nanotubes, graphene materials, mesoporous carbon, quantum dots, noble metal nanoclusters, metal, and metal oxide nanoparticles [25,26,27,28,29,30,31]. Inspired by the potential generating and transducing optical characteristics, in the recent past, nanomaterials-based colorimetric sensors have been largely explored with excessive selectivity, sensitivity, and response time [26,30,32,33] through acknowledging the changes in various photophysical response following interaction with metal ions. Furthermore, the colorimetric sensing approach complements the barriers in the utilization of expensive instruments, complex sample preparation, and employing trained personnel. While these nanomaterials-based colorimetric detection give rise to monitoring the metal ions, special emphasis is laid on incorporating such nanomaterials in a suitable solid platform to overcome the obstacle for enabling their real-time and onsite applications.

**Figure 1:**
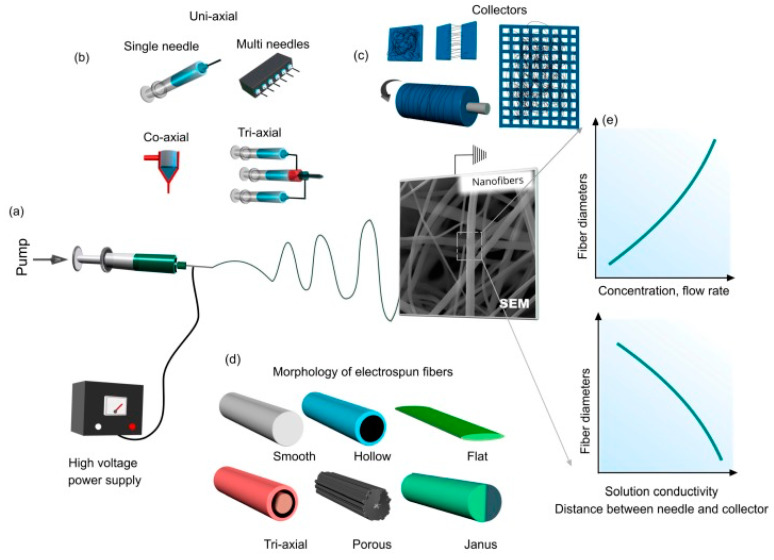
An electrospinning setup with important parameters is shown. (**a**) A cartoon scheme of an electrospinning system with the scanning electron micrograph of electrospun fibers, (**b**) common spinneret systems used in electrospinning, (**c**) collector types, (**d**) the morphology of electrospun fibers, and (**e**) diagrams showing the influence of electrospinning process parameters and solution properties on the electrospun fibers. Reprinted from [37], 2019, MDPI.

Electrospinning is a facile technique for producing ultrafine/nanofibers from a variety of materials through the process of pumping a viscous solution through a needle under high voltage, and when the value of electrostatic repulsion overcomes the surface tension of the solution, a continuous jet is formed from the pendant drop that further undergoes spiraling, stretching, and solidification upon solvent evaporation; then, finally, nonwoven fibers are deposited on the collector [34,35,36]. Electrospinning adopts several components, parameters, and processes in the fabrication of electrospun nanofibers with different alignments, diameters, and morphology, as can be seen from Figure 1 [37]. Several research efforts have been pioneered from the excellent features of electrospun nanofibers such as a high aspect ratio, large specific surface area, tunable porosity, interconnectivity, easy functionalization, flexibility, easy handling, and mechanical stability for their applications in energy [38,39,40,41,42], environmental [38,39,40,43,44,45,46,47,48,49,50,51,52], electronics [53,54,55], and biomedical sectors [39,40,56,57,58,59,60,61,62,63,64,65,66]. Especially, the electrospun nanofibers have spectacularly benefited from the design and fabrication of sensing platforms for the colorimetric detection of metal ions with high sensitivity and quick response through various means of functionalization, mainly through direct blending and the surface immobilization of functional agents for metal ion interaction [49,52,67,68,69,70]. Here, we review notable achievements that have harnessed the design of functionalized electrospun nanofibers for the colorimetric detection of various metal ions such as copper, mercury, lead, cadmium, chromium, iron, zinc, nickel, cesium, aluminum, and silver. This review critically highlights the progress on designing the functionalized electrospun nanofibers for the colorimetric detection of metal ions and provides an outlook for the future exploration.

## 2. Designing of Functionalized Electrospun Nanofibers for the Colorimetric Detection of Metal Ions

### 2.1. Detection of Copper Ions

**Figure 2:**
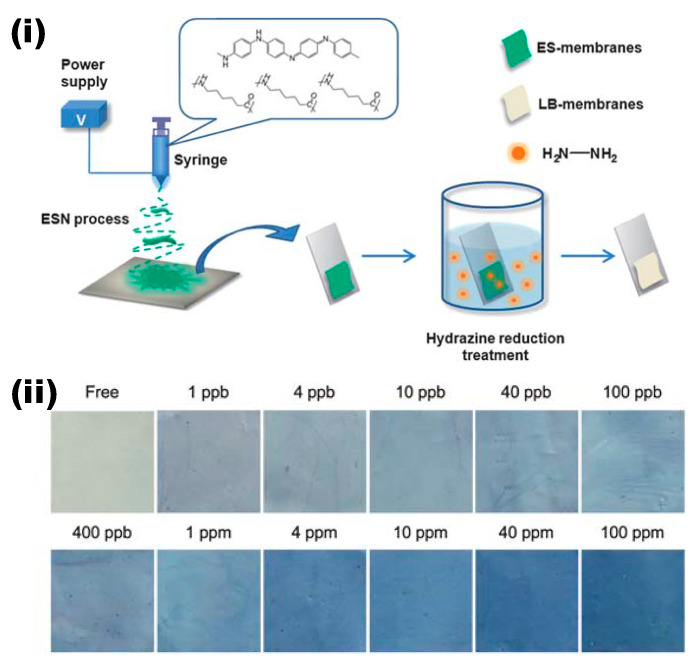
(**i**) Representation of the preparation of the polyaniline/polyamide-6 (PANI/PA-6) colorimetric nanofiber/net (NFN) membranes. (**ii**) Optical colorimetric response of the sensor strips after incubation for 30 min in Cu^2+^ aqueous solutions with different concentrations. Reprinted with permission from [71], 2011 © The Royal Society of Chemistry.

Copper (Cu^2+^) is one of the essential elements required for several biological processes in the human body; however, its excessive exposure leads to damaging several organs and tends to cause Alzheimer’s disease, Menkes disease, Wilson’s disease, and Parkinson’s disease. So far, accountable progress has been pioneered in developing electrospun nanofiber systems for the colorimetric detection of Cu^2+^ ions. Table 1 summarizes the corresponding studies proceeded for the colorimetric detection of Cu^2+^ ions by employing different polymeric materials, functional molecules, and approaches along with their performances [71,72,73,74,75,76,77,78,79,80,81,82,83,84,85,86,87,88,89,90,91]. An early seminal work in the vein of developing electrospun nanofibers for the colorimetric sensing of Cu^2+^ ions and conjugated polymers complemented from their potential beneficial characteristics associated with delocalized π-network and conformationally restricted polymer chains has been used in the fabrication of a nanofiber-based sensor for the detection of Cu^2+^ ions [71]. A flexible sensor strip of polyaniline/polyamide-6 (PANI/PA-6) nanofiber/net (NFN) membranes have been fabricated by dissolving a synthesized PANI emeraldine base (PANI-EB) and PA-6 followed by electrospinning to obtain a green NFN membrane. Furthermore, the obtained membranes were fixed on a glass slide and treated with hydrazine (40 wt % aqueous solution) to get colorimetric membranes, which comprise the PANI leucoemeraldine base (PANI-LB) and PA-6 (LB-membranes). Figure 2i depicts the schematic representation of the typical procedure for fabrication of the PANI/PA-6 NFN membranes. The sensing experiments were performed by immersing 15 mm × 15 mm square strips of PANI/PA-6 colorimetric NFN membranes in the Cu^2+^ ion solutions, and the spectral changes were measured by an optic spectrometer. Furthermore, a qualitative color change was observable with the naked eye, and quantitative estimation was performed using the converting method, which converts the reflectance spectrum into red, green, and blue (RGB) values. Upon exposure to various concentrations ranging from 1 to 100 ppm, decreases in the two main intensity bands at 650 and 435 nm were noted. The colorimetric response of the sensor strip toward various concentrations of the sensor strips is depicted in Figure 2ii. The sensor strip displayed a typical color change from white to blue upon an increase in Cu^2+^ concentration with a detection limit of 1 ppb. Furthermore, the sensor strip demonstrated high selectivity over a variety of common metal ions [71].

In another clever example, a naphthalimide-functionalized nanofibrous film was prepared by a copolymerization and electrospinning approach [72]. Briefly, the sensing material poly (MMA-co-NAAP) has been synthesized by the copolymerization of 1,8-naphthalimide functionalized (NAAP) with methyl methacrylate, and further, the poly (MMA-co-NAAP) was dissolved in *N,N*-Dimethylformamide (DMF) at a concentration of 25 wt %, after which it was electrospun to obtain the sensing membrane. The fluorescence behavior of the nanofibrous film was evaluated by exposing to Cu^2+^ at the concentration ranging from 0 to 20 µM, and the corresponding results showed a blue shift in the emission range of 487 to 439 nm, which could be attributed to the deprotonation of the secondary amine conjugated to the naphthalene ring. The highest sensitivity of the sensor film was determined as 2.0 × 10^−5^ M. Furthermore, the sensing film demonstrated the sensitivity over three months, and the nanofibrous sensor film showed excellent selectivity toward Cu^2+^ over several common metal ions [72]. Similarly, Wu and Lai adopted a free radical copolymerization process for the preparation of poly[(N-isopropylacrylamide)-*co*-(N-hydroxymethyl acrylamide)-*co*-(4-rhodamine hydrazonomethyl-3-hydroxy-phenyl methacrylate)] [poly(NIPAAm-*co*-NMA-*co*-RHPMA), PNNR] using three monomers such as thermo-responsive *N*-Isopropylacrylamide (NIPAAm), chemically crosslinkable *N*-hydroxymethylacrylamide (NMA), and Cu^2+^ sensing 4-rhodamine hydrazonomethyl-3-hydroxy-phenyl methacrylate (RHPMA), which has been further used for electrospinning to obtain nanofibers [73]. For the purpose of comparison, polymer films with similar concentration have been prepared through the dip-coating method. The sensing experiment has been carried out by taking advantage of the ring-opening process of rhodamine fluorochrome. In a typical experimental process, RHPMA moieties on the prepared copolymers are first activated in an acidic environment to exhibit strong fluorescence and pink color; further quenching of the fluorescence has been monitored following exposure to Cu^2+^ through the “ON–OFF” sensing mechanism. The corresponding results indicated that the electrospun nanofibers displayed a much higher sensing performance compared to thin films, owing to their higher surface area. Furthermore, the nanofibers showed good selectivity toward Cu^2+^ in the presence of other competitive metal ions. In addition, the PNNR nanofibers also shown on/off switchable sensing characteristics with response to temperature change attributed to the hydrophilic–hydrophobic transition of PNIPAAm [73]. An identical copolymerization and electrospinning approach with various functional sensing moieties have also been adopted for the development of electrospun nanofiber-based colorimetric sensors to detect the Cu^2+^ [74,75].

**Figure 3:**
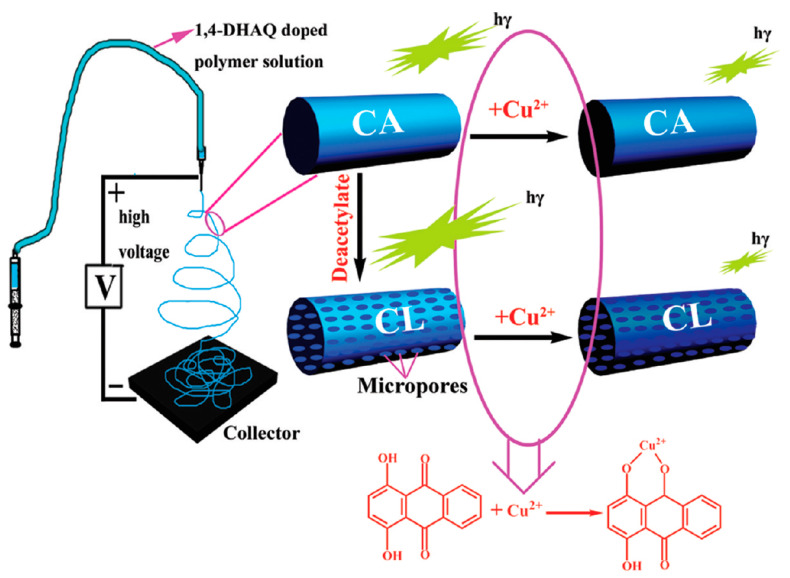
Schematic showing the electrospinning setup (left) and sensing mechanism of the 1,4-dihydroxyanthraquinone with cellulose (1,4-DHAQ@CL) microporous nanofiber film for Cu^2+^ (right). Reprinted with permission from [76], 2012 © American Chemical Society.

Starting from some key findings, further efforts also paved toward the direct incorporation of various functional molecules in the electrospinning solution for the fabrication of colorimetric Cu^2+^ sensors. In this context, a microporous electrospun nanofiber (1,4-DHAQ@CL) has been produced through the doping of 1,4-dihydroxyanthraquinone (1,4-DHAQ, a fluorophore) with cellulose (CL) [76]. The rigid backbone of 1,4-DHAQ tends to interact with phenyl groups on the CA side through π−π stacking, thus limiting the aggregation of the fluorophores. The schematic representation of the fabrication electrospun nanofibers and their sensing mechanism toward Cu^2+^ is illustrated in the Figure 3. In an initial step, 1,4-DHAQ doped cellulose acetate (CA) nanofiber film has been fabricated and further turned into 1,4-DHAQ@CL through the deacetylation process. The deacetylation treatment increases the exposure of 1,4-DHAQ fluorophore molecules on the nanofiber surface, which offers enhanced interaction with Cu^2+^ and also improves the sensitivity. The sensing experiments demonstrated that the fabricated 1,4-DHAQ@CL showed a steady quenching behavior toward Cu^2+^ with the detection limit of 3 nM. The sensing mechanism could be explained by the formation of Cu^2+^-based phenolate with 1,4-DHAQ. The selectivity experiments with several common metal ions including Ni^2+^, Cd^2+^, Pd^2+^, Hg^2+^, Cu^2+^, Cr^2+^, Zn^2+^, Na^+^, K^+^, Al^2+^, Mg^2+^, Ca^+^, Co^2+^, and Fe^2+^ showed very good selectivity to Cu^2+^ even in the presence of a 100-fold higher concentration of ions other than the Cu^2+^. In addition, the sensor film was also found to have excellent reusable characteristics following simple treatment [76].

Inspired by the signal switching characteristics of the spirolactam moiety of the rhodamine group upon interaction with cations, the rhodamine dyes are also used to incorporate in the polymeric matrix and were explored for their possibilities in the sensing of Cu^2+^. As evidence, rhodamine dye-doped electrospun poly(ether sulfones) (PES) nanofibers have been fabricated and tested for their competence in Cu^2+^ sensing [77]. In a typical experimental process, the rhodamine dye was synthesized using a two-step process and then incorporated into PES polymeric solution; then, it was electrospun to obtain a rhodamine dye-doped PES nanofibrous sensor. The emission behavior of the sensor treated with Cu^2+^ has been recorded, and the corresponding observation indicated an increased absorption intensity behavior at approximately 560 nm, and simultaneously, the color of the sensor significantly turned from white to pink due to Cu^2+^-induced formation of a ring opening in the spirolactam moiety. The developed sensor has the lowest detection limit of 1.1 × 10^−9^ M, has excellent selectivity over other common interfering ions, and possesses excellent reusability characteristics up to 10 cycles following treatment with tetrasodium ethylene-diamine tetraacetic (Na_4_EDTA) [77]. Similarly, Tungsombatvisit et al. fabricated rhodamine B derivative (RBD)-doped cellulose acetate (CA) electrospun nanofibers and demonstrated their potency in Cu^2+^ sensing in water [78]. Briefly, the RBD has been synthesized first and then incorporated into CA polymeric solution followed by electrospinning to produce the sensing strip and further made alkaline treatment. The alkaline treatment resulted in partial deacetylation, which reduced the number of acetyl groups while increasing the hydroxyl groups in the fibrous structure, offering enhanced wettability. The sensor strip has been immersed in the various concentrations of Cu^2+^ solution to establish their detection limit, which was found to be 18 ppm. The alkaline treatment of the nanofibers offered 33 times faster response than the untreated one [78].

In addition to these developments, continuous efforts have been also made in to utilize fluoroinophores in the Cu^2+^ sensing. For instance, the electrospun nanofibers of poly(methyl methacrylate) (PMMA) or ethyl cellulose (EC) incorporated with fluoroinophore, N’-3-(4-(dimethylamino phenly)allylidene)isonicotinohydrazide (DPAINH)) have been used for Cu^2+^ sensing [79]. The DPAINH has been selected owing to their selective response to Cu^2+^. The resultant sensor membrane demonstrated good sensitivity toward Cu^2+^ and their limit of detection was determined as 3.8 × 10^−14^ M and 1.4 × 10^−13^ M for EC and PMMA membranes. Furthermore, the electrospun nanofibers demonstrated improved performance compared with the thin films prepared with similar formulation and also demonstrated good selectivity over other possible interferents [79]. Another study reported the use of an ultrasound-electrospinning approach for the fabrication of a polyvinyl alcohol (PVA)/tetraethyl orthosilicate (TEOS)/Schiff base, which was used in the detection of Cu^2+^. Initially, the Schiff base has been sonochemically prepared and immobilized with PVA and TEOS through an electrospinning approach. The fabricated sensor showed excellent Cu^2+^ sensing performance with a detection limit of 1.27 × 10^−8^ mol L^−1^ and also showed excellent selectivity [80]. 

**Figure 4:**
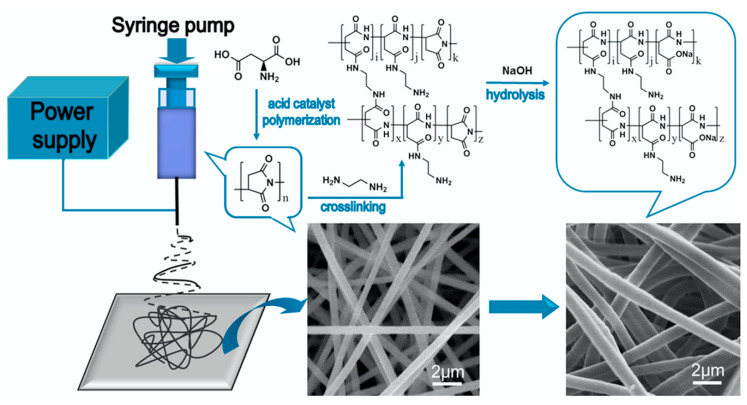
Representation of the preparation of the poly (aspartic acid) nanofibrous hydrogel (PASP-NH). Reprinted with permission from [81], 2015 © Elsevier.

In another study, a biodegradable colorimetric chemosensor for the detection of Cu^2+^ has been prepared using poly (aspartic acid) (PASP) nanofibrous hydrogel with enhanced hydrophilicity through adopting the complexation phenomena of poly (aspartic acid) nanofibrous hydrogel and the color changing feature attributed to biuret reaction [81]. As a first step, polysuccinimide (PSI) as an intermediate of PASP has been synthesized through the polymerization of aspartic acid, and it was dissolved in *N, N*-Dimethylformamide (DMF) and then electrospun into nanofibers followed by crosslinking with ethylenediamine and hydrolyzation to obtain PASP nanofibrous hydrogel (PASP-NH) (see Figure 4). The prepared PASP-NH has been further employed in the colorimetric Cu^2+^ sensing, and the corresponding results demonstrated the color of PASP-NH changes from white to purple and then violet upon increasing the Cu^2+^ concentration, and the detection limit was determined as 0.01 mg/L. The PASP-NH membrane has a large surface area and abundant interconnected pores that not only boost analyte diffusion but also enhance the interaction of analytes on the surface of PASP-NH. The sensing phenomenon includes chemical sorption and biuret reaction. The selectivity experimental observation showed good response over several common metal ions and also good reversibility. In a recent similar effort, Zhang et al. also developed a PASP electrospun nanofiber hydrogel membrane (PASP−ENHM) that was used in Cu^2+^ sensing, and the corresponding colorimetric sensing outcome revealed a good response toward Cu^2+^ with the naked eye detection limit of 0.3 mg/L [82].

**Figure 5:**
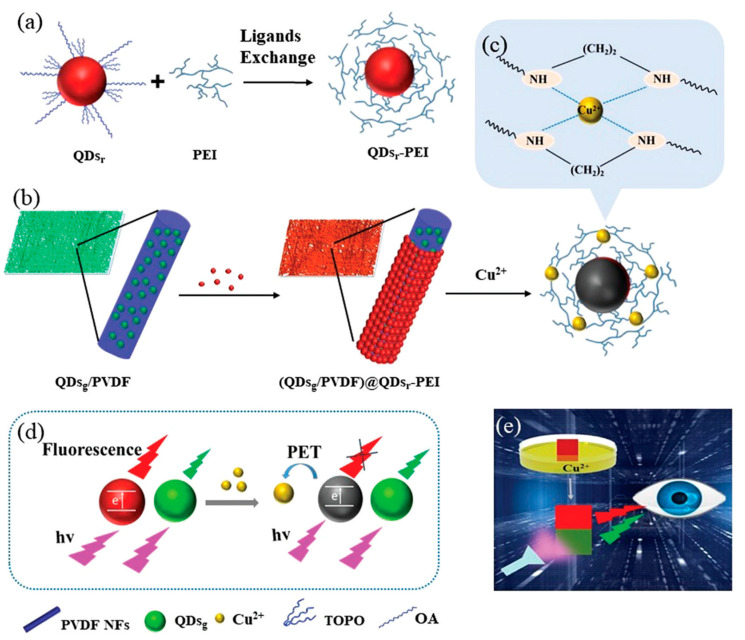
Schematic diagram for the preparation of dual fluorescent (QDsg/PVDF)@QDsr-PEI membranes (**a**,**b**). Detection mechanism and change in the fluorescence of QDs following interaction with Cu^2+^ (**c**,**d**). Cartoon representation of visual color change (**e**). Reprinted with permission from [84], 2019©The Royal Society of Chemistry.

In recent years, owing to the enhanced optical characteristics, the incorporation of quantum dots-based electrospun nanofiber sensing platforms has drawn considerable interest in the detection of Cu^2+^. As an example, CsPbBr_3_ perovskite quantum dots (CPBQDs) have been encapsulated in nanoscale polymethyl methacrylate (PMMA) fiber membrane (CPBQDs/PMMA FM) through electrospinning; then, they were functionalized with cyclam and used in fluorescence resonance energy transfer (FRET) detection of Cu^2+^ [83]. Benefiting from the excellent optical characteristics and subsurface concentration of the CPBQD in the PMMA FM and their surface area, the developed sensor showed outstanding sensitivity and stable detection performance over the thin film with a detection limit of 10^−15^ M. Another recent investigation demonstrated a dual-fluorescent sensor for the real-time and visual detection of Cu^2+^ [84]. In a typical experimental procedure as a first step, green-emitting quantum dots (QDs_g_) and red-emitting quantum dots (QDs_r_) have been synthesized and furthermore, the QDs_r_ has been modified with polyethylenimine (PEI) (QDs_r_-PEI) for the purpose of improving sensitivity toward Cu^2+^ detection because of the high Cu^2+^ binding of PEI due to the electron-rich amino groups. The QDs_g_ and poly(vinylidene fluoride) (PVDF) has been dissolved in dimethylformamide (DMF) and acetone; then, they were further electrospun to obtain a QDsg/PVDF membrane. Then, the electrospun QDsg/PVDF membrane was coated with the QDs_r_-PEI to obtain dual fluorescent (QDsg/PVDF)@QDs_r_-PEI membranes and used for Cu^2+^ detection. Figure 5 represents the preparation process and sensing mechanism of dual fluorescent QDsg/PVDF)@QDs_r_-PEI membranes. The sensing experiments showed that the interaction of Cu^2+^ with QDs_r_-PEI in the outer layer of the PVDF membrane resulted in an obvious fluorescence quenching with a fast response time of 30 s and detection limit of 2 µM. Furthermore, the selectivity experiments showed excellent response over various cations including Ca^2+^, Zn^2+^, Cd^2+^, Na^+^, K^+^, Pb^2+^, Hg^2+^, Cu^2+^, Ag^+^, Fe^3+^, Mg^2+^, and common anions including Cl^−^, NO3^−^, and SO_4_^2−^ at a concentration of 100 µM [84].

**Figure 6:**
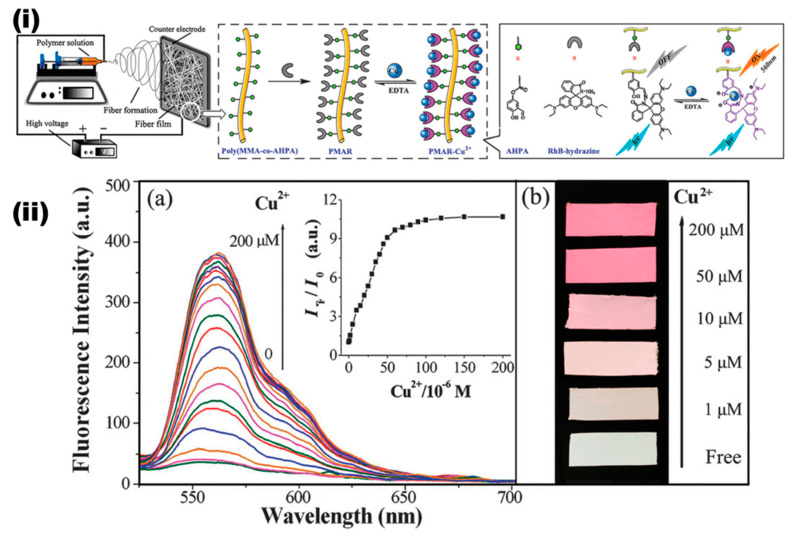
(**i**) Chemical and schematic illustration of the preparation of nanofibrous film fluorescent sensors for Cu^2+^ ions. (**ii**) (**a**) Fluorescent spectra of the PMAR nanofibrous film in the absence and presence of Cu^2+^ (1.0 × 10^−6^–2.0 × 10^−4^ mol L^−1^). The inset shows fluorescence intensity change as a function of Cu^2+^ concentration. (**b**) From the bottom to top are photographs of the nanofibrous film after 1 mM, 5 mM, 10 mM, 500 mM, and 200 mM Cu^2+^ involvements. Reprinted with permission from [85], 2013 © The Royal Society of Chemistry.

While these developments have their own breakthroughs in the development of a Cu^2+^ colorimetric sensor, further approaches on grafting functional moieties on the surface of the electrospun nanofibers brought the Cu^2+^ sensing domain a step forward. In an early study, Wang et al. used the covalent grafting approach to functionalize the rhodamine derivative functional molecules on the nanofiber surface to establish high efficiency through changing the surface properties including charge and hydrophilicity [85]. In a typical experimental procedure, rhodamine B-hydrazine (RhB-hydrazine) and 4-aldehyde-3-hydroxy phenyl acrylate (AHPA) have been synthesized; furthermore, the free radical copolymerization of AHPA and methyl methacrylate (MMA) has been performed in an anhydrous solution of *N,N*-Dimethylformamide (DMF) with an initiator 2,2-azobis(isobutyronitrile) (AIBN). The resultant was further used to prepare a poly (MMA-*co*-AHPA) nanofibrous film that possesses a large amount of aldehyde groups on the surface. Further, Rh6B-hydrazine fluorophores were grafted on the surface of the nanofiber (PMAR) by allowing the reaction between the amino groups of fluorophore and aldehyde groups, which was further used in the sensing application. The preparation procedure and the “turn-on” sensing performances are schematically provided in Figure 6. The sensing efficiency of the prepared PMAR demonstration by turning the white color membrane into pink upon interaction with Cu^2+^ by chelating imine N, carbonyl O, and phenol O atoms of the fluorophore moiety resulted in a significant enhancement of fluorescence at 557 nm, as shown in Figure 6ii. The detection limit of the PMAR was determined as 1.5 × 10^−6^ mol L^−1^ within a 10 s time frame. Furthermore, the selectivity experiments over several competitive cations lead to no significant color enhancement, and also, the membrane possesses an excellent reusability feature following treatment with ethylenediaminetetraacetic acid (EDTA) solution [85].

A similar effort has been cemented toward the preparation of pyrene derivative 2-((pyren-1-yl) methyleneamino)-3-amino maleonitrile) (PyDAN2)–modified poly (2-hydroxyethyl methacrylate-*co*-N-methylolacrylamide) (poly(HEMA-*co*-NMA)) electrospun nanofibers for the highly selective detection of Cu^2+^ with a detection limit of 10^−7^ to 10^−6^ M [86]. Cui et al. also demonstrated the fabrication of ethylene-vinyl alcohol copolymer (EVOH) electrospun nanofibers and their functionalization with 4-aminobenzoic acid (PABA) and 1-pyrenecarboxaldehyde (Py-CHO) for the purpose of selective and fast Cu^2+^ detection, and the results showed good sensing response [87]. Another approach has been implemented by functionalizing carbon nanotubes (CNTs) on 1,4-dihydroxyanthraquinone (1,4-DHAQ)-doped microporous cellulose nanofibrous membrane ((1,4-DHAQ doped CNTs@CL) [88]. The CNTs’ modification offers excellent sensitivity and selectivity, which is attributed to their outstanding adsorbability and the collection capacity of metal ions. The sensing behavior showed that the characteristics peak at 534 nm, which decreases upon interaction with Cu^2+^ with a detection limit of 2.17 × 10^−9^ M. Furthermore, the prepared sensing membranes showed excellent selectivity over several common interfering ions [88].

In the recent years, substantial progress was also made on the decoration of noble metal nanoparticles on the surface of electrospun nanofibers and extended their applications in sensing of Cu^2+^. In one specific example, Abedalwafa et al. described the fabrication of a colorimetric sensor strip using aminated polyacrylonitrile (APAN) electrospun nanofibers carrying gold/silver core/shell nanoparticles (Au/Ag NPs) [89]. As a first step, polyvinylpyrrolidone (PVP)/polyacrylonitrile (PAN) composite electrospun nanofibers have been prepared and extracted in water to remove PVP; as a result, a rough surface has been obtained and further modified with ethylenediamine (EDA). Simultaneously, the prepared core/shell Au/Ag NPs have been immobilized on the surface of nanofibers through covalent bonds and used in sensing experiments. The sensing experiments revealed that the wavelength measured at 420 nm has been red shifted upon exposure to Cu^2+^. Furthermore, the sensor strip color has been turned from yellow to pink to colorless, owing to the leaching of Au/Ag NPs in the presence of ammonium chloride, thiosulfate, and Cu^2+^, which then forms thiosulfate complexes. The detection limit of the prepared sensor strips was noted as 50 nM in a 3 min response with excellent specificity and reversibility [89]. 

**Figure 7:**
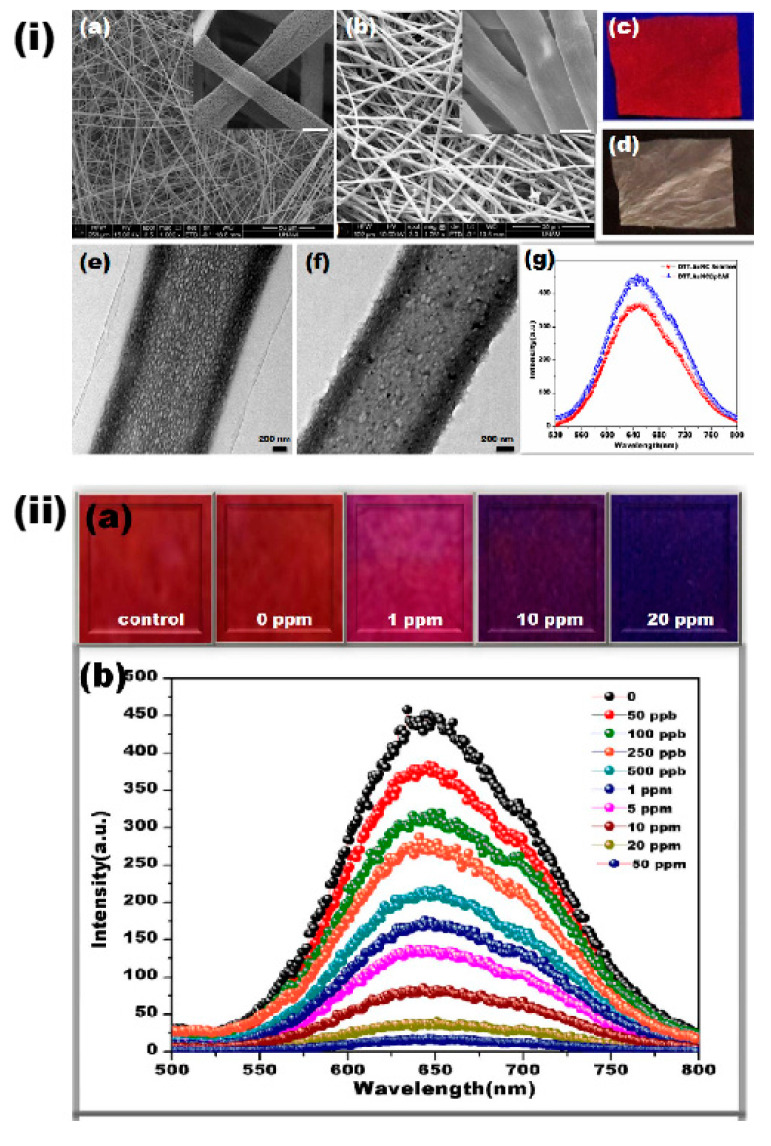
(**i**) (**a**) SEM images of the electrospun porous cellulose acetate fibers (pCAF) before and (**b**) after incorporating dithiothreitol capped gold nanocluster (DTT.AuNC). The higher magnification view shows as an inset in their corresponding figures (scale bar: 1 μm). (**c**) Photograph of the DTT.AuNC@pCAF under UV light (λ_ext_-254 nm) and (**d**) day light condition. TEM image of single DTT.AuNC@pCAF (**e**) in the presence and (**f**) absence of excess DTT ligand. (**g**) Compared emission spectra of DTT.AuNC solution and DTT.AuNC@pCAF. (**ii**) (**a**) Visual colorimetric detection of Cu^2+^. A clear difference in the color from red to blue is noticed under UV light with an increasing concentration of Cu^2+^. The photograph has been taken under exposure of UV light (λ_ext_-254 nm). (**b**) Fluorescence spectra of DTT.AuNC@pCAF upon exposure to various concentration of Cu^2+^ in water. Reprinted from [90], 2015, Springer Nature.

While the characteristics of the noble metal NPs are appreciated in the sensing applications, their protection in nanofibers architecture is critical and imparts improved performances. To reveal the phenomenon, Senthamizhan and co-workers developed an elegant method of fabricating highly selective and sensitive detection sensors using vastly porous cellulose acetate fibers (pCAF) with dithiothreitol capped gold nanocluster (DTT.AuNC) as a fluorescent probe [90]. In a detailed description, pCA and normal cellulose acetate (nCA) has been prepared and coated with the DTT.AuNC to obtain DTT.AuNC@pCAF and DTT.AuNC@nCAF, respectively. Figure 7i,ii demonstrates the morphological feature and sensing performances of the DTT.AuNC@pCAF. A careful optimization investigation for improving the sensor’s performance has been conducted especially for the DTT.AuNC coating and the removal of excess ligand on the nanofiber surface (see Figure 7i,e,f) thus helped in controlling the aggregation of AuNC, which a plays critical role in the analyte interaction. Moreover, each pore in the pCA has been acted as a cavity that actually protected the AuNC and the effective encapsulation not only enhanced the sensing performance but also improved the stability against environmental factors. As an evident, the prepared DTT.AuNC@pCAF has been found stable for a period of more than six months with no significant detachment of DTT.AuNC and change in coating density, but this is not the case with the DTT.AuNC@nCAF. The anchoring of DTT.AuNC on pCA has been achieved through the formation of hydrogen bonding between the thiol (-SH) or hydroxyl group in DTT with an acetate group present in CA. In the sensing experiment, an effective contact mode has been adopted to expose the sensor membrane in various Cu^2+^ concentrations, and the results revealed a uniform color change from red to blue, as can be seen from the Figure 7iia. The corresponding spectral changes indicated a gradual quenching of emission upon increasing the concentration (see Figure 7iib) with the detection limit of 1 ppm (visual) and 50 ppb (spectroscopy). The improved sensing performance benefited from the porous structure increased the analyte diffusion inside the fibers and uniform distribution of the DTT.AuNC supplements’ reactive sites. The sensing mechanism was attributed to the formation of aggregated Au–Cu blend owing to the tendency of Cu to complex with the free thiol group and oxidation of DTT, thus resulting in the reduction of energy transfer from the gold core to the metal ligand complex. Further, interference studies revealed a selective sensing performance of the DTT.AuNC@pCAF against various metal ions, including Hg^2+^, Cd^2+^, and Zn^2+^. The findings of the study revealed several advantages including selective detection, the high stability that is required for outdoor applications, no formation of toxic products, and easy handling and disposal [90]. Another approach has witnessed the sensing of Cu^2+^ using nitrogen-doped carbon dots (N-CDs) derived from the chemical breakdown of electrospun polyacrylonitrile (PAN)-based carbon nanofibers with the detection limit of 5 nM [91]. Briefly, the PAN electrospun nanofibers have been fabricated and further subjected to carbonization process; then, the carbonized fibers have been treated with nitric acid and dialyzed for three days in neutral pH to obtain N-CDs, which were further used in the sensing performance. The sensing studies revealed the quenching of emission intensity at 510 nm ascribed from the phenomenon that the coordination interaction of the Cu^2+^ with N and O containing groups of the N-CDs, thus leading to nonradiative electron transfer or energy transfer [91].

### 2.2. Detection of Lead Ions

**Figure 8:**
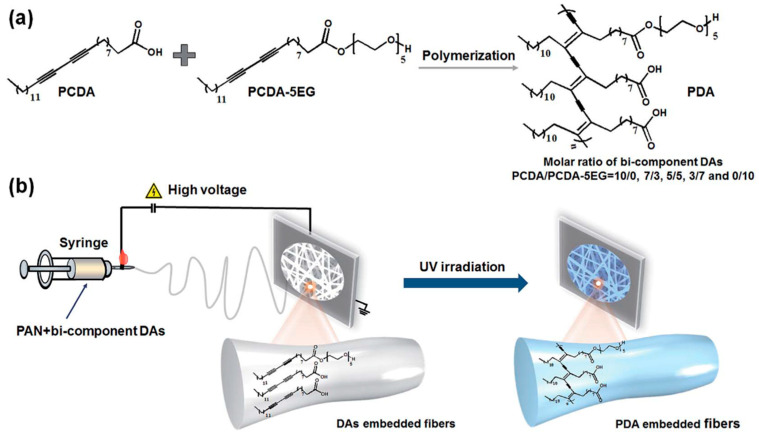
(**a**) Self-assembly and polymerization of 10,12-pentacosadiynoic acid (PCDA) and the pentaethylene glycol headgroup (PCDA-5EG). (**b**) Schematic representation of the preparation of the PDA embedded polyacrylonitrile nanofibrous membrane (PAN NFM). Reprinted with permission from [92], 2014 © The Royal Society of Chemistry.

Lead (Pb^2+^) is a persistent environmental pollutant that possess long-lasting environmental and human risks. The increased levels of Pb^2+^ lead to several severe complications in the human body including damages to the kidney, liver, nervous, and cardiovascular system. Especially, the trace amounts of Pb^2+^ in blood cause decreased intelligence and permanent behavioral disorder. In addition to the several progresses made on the detection of Pb^2+^, efforts to design efficient electrospun nanofiber-based colorimetric sensors have been driven. Table 2 summarizes the corresponding studies proceeded for the colorimetric detection of Pb^2+^ by employing different polymeric materials, functional molecules, and approaches along with their performances [92,93,94,95,96,97,98,99]. In this aspect, Li et al. demonstrated the fabrication of using polydiacetylene (PDA) embedded polyacrylonitrile nanofibrous membrane (PAN NFM) [92]. Briefly, PDA has been prepared from the mixtures of 10,12-pentacosadiynoic acid (PCDA) and PCDA derivative with a pentaethylene glycol headgroup (PCDA-5EG). The electrospun nanofibers have been prepared by electrospinning the precursor solution containing bi-component DAs (PCDA and PCDA-5EG) and PAN. The resultant electrospun nanofibers have been stored in the dark, and further photopolymerization has been performed by exposing the Das-embedded nanofibers in UV irradiation at 254 nm, which turns them into a PDA-embedded membrane with a blue color. The preparation procedure of the sensor strip is schematically depicted in Figure 8. The sensing response of the prepared membrane has shown an obvious blue-to-red transition upon the addition of Pb^2+^ with a decrease of the absorption at 645 nm and the simultaneous increase of absorption at 550 nm with a detection limit of 0.48 µM. The color change was promoted by the ligand–receptor reaction between Pb^2+^ and the strips [92]. A similar demonstration has been performed for the fabrication of pH-paper-like Pb^2+^ sensor using the SiO_2_ nanoparticle (NP) decorated polydiacetylene contains glycine (Gly) in its headgroup embedded polyacrylonitrile nanofibrous membrane (PAN NFM) [93].

To date, substantial progress has been also made in the fabrication of curcumin (CC)-based cellulose acetate (CA) nanofibers [94]. In a typical experimental process, CC was dissolved in CA polymeric solution, and then, the solution was electrospun to obtain CC-CA electrospun nanofibrous sensor strip for the detection of Pb^2+^. The fabricated CC-CA nanofibers were exposed to various concentrations of Pb^2+^ and the corresponding results indicated that a yellow to orange color change has been noted with the detection limit of 0.12 ± 0.01 µM due to the complex formation between the curcumin and Pb^2+^. The selectivity experiments further showed an excellent response only toward Pb^2+^ not with any other meta ions including Ba^2+^, Ca^2+^, Co^2+^, Cd^2+^, Cu2+, Mg^2+^, Ni^2+^, and Zn^2+^ [94]. Another similar investigation used curcumin nanoparticles (CC NPs) impregnated CA nanofibers for the detection of Pb^2+^ and the corresponding results revealed the visual and linear graph detection limit of 0.4 mM and 0.14 ± 0.01 mM, respectively [95].

**Figure 9:**
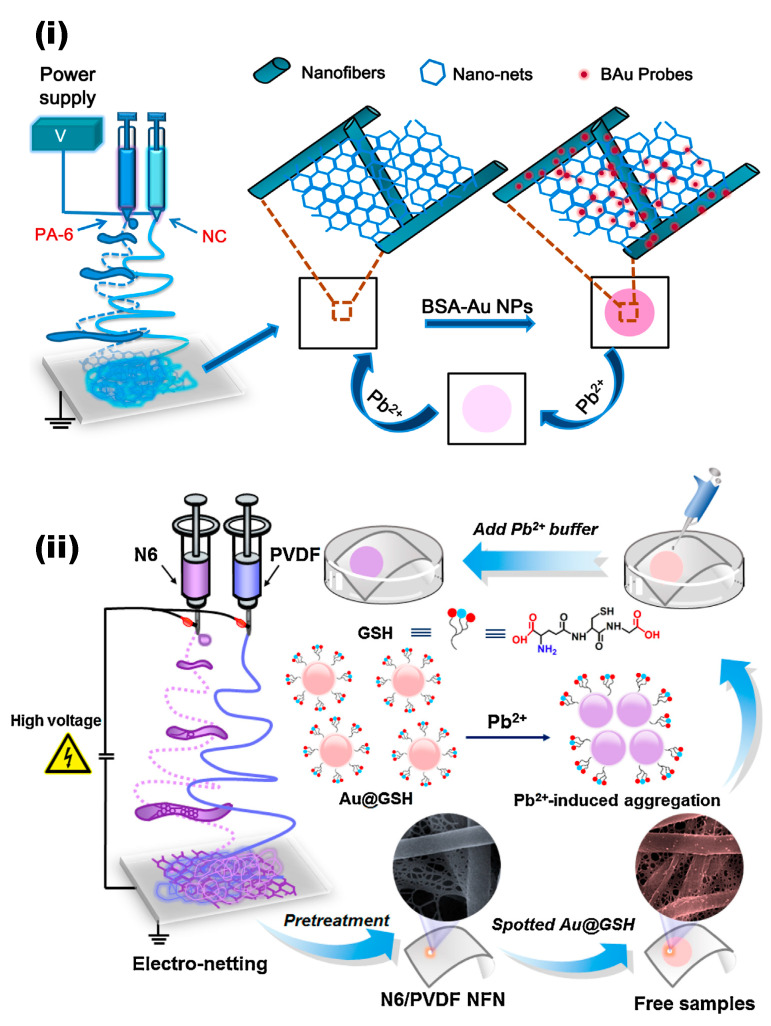
(**i**) Representation of the preparation of the bovine serum albumin-decorated Au nanoparticles (BAu probe) immobilized polyamide-6/nitrocellulose (PA-6/NC) colorimetric strips. Reprinted with permission from [96], 2013 © Elsevier. (**ii**) Schematic illustration of the configuration and measurement principle of the L-glutathione-conjugated Au nanoparticle probes (Au@GSH) immobilized nylon-6/polyvinylidene fluoride (N6/PVDF) chromatic sensor strips. Reprinted with permission from [97], 2014 © Elsevier.

The functionalization of gold nanoclusters on the surface of electrospun nanofibers is another ideal strategy for the ultrasensitive detection of Pb^2+^. In this context, early work by Li and co-workers addressed the preparation of sensors utilizing electrospun polyamide-6/nitrocellulose (PA-6/NC) nanofibers/nets (NFN) membranes assembled with bovine serum albumin-decorated Au nanoparticles (BAu probe) through the multi-jet electrospinning approach for Pb^2+^ detection [96]. The resultant fibers possess 2D spider web-like nano-nets with 3D porous structures. The first prepared BAu probe has been synthesized and dip coated on the PA-6/NC NFN membranes, as shown in the Figure 9i. The sensing experiments have demonstrated a visual color change from deep pink to white and a significant absorption decrease in band at 546 nm following exposure to Pb^2+^ solution with a detection limit of 0.2 µM has been noted, which was much higher than that of the film-based ones. The observed responses were attributed the Pb^2+^ ions accelerating the leaching of Au NPs with the induction caused by thiosulfate and 2-mercaptoethanol, which leads to a noticeable color change [96]. In another similar example, L-glutathione-conjugated Au nanoparticle probes (Au@GSH) assembled electrospun nylon-6/polyvinylidene fluoride (N6/PVDF) nanofibers/nets (NFN) membranes for the high-throughput determination of Pb^2+^ have been prepared through the process depicted in Figure 9ii [97]. The sensing experiment demonstrated Pb^2+^ induced Au@GSH aggregation that triggered a color change from pink to purple. The developed sensor demonstrated Pb^2+^ sensing performances with a detection limit of 10 µg/dL in a rapid response time (10 min) [97].

**Figure 10:**
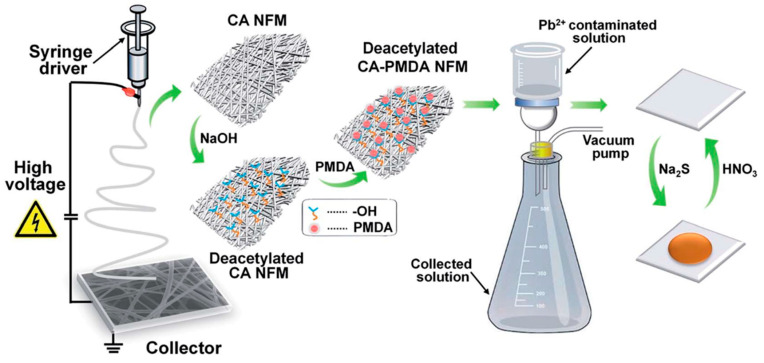
Schematic illustration of the fabrication procedure of deacetylated cellulose acetate nanofibrous membranes with pyromellitic dianhydride (DCA-PMDA NFM) and the simultaneous colorimetric detection and enrichment of the target Pb^2+^ by DCA-PMDA NFM. Reprinted with permission from [98], 2015 © The Royal Society of Chemistry.

Li et al. used the affinity features of pyromellitic dianhydride (PMDA) and structural merits of electrospun nanofibers; a Pb^2+^ sensor has been fabricated thorough the modification of deacetylated cellulose acetate (DCA) membranes with PMDA (DCA-PMDA) [98]. Figure 10 demonstrates the preparation process and colorimetric Pb^2+^ detection. As an initial step, the precursor polymeric solution of CA has been electrospun to obtain a CA nanofibrous membrane (CA NFM), which has been further subjected to the deacetylation process. Furthermore, the deacetylated CA NFM has been grafted with PMDA and used in a colorimetric Pb^2+^ homemade poly-(methyl methacrylate) flow cell. The colorimetric detection has been performed by treating the Pb^2+^ solution filtered DCA-CA NFM with sodium sulfide (Na_2_S), which results in a formation PbS. The results indicated that the prepared sensor efficiently detected Pb^2+^ with a detection limit of 0.048 µM [98]. In another study, Zhang et al. adopted a plasmon-enhanced fluorescence (PEF) activity concept in demonstrating the general and facile approach for the fabrication of polyacrylonitrile (PAN)/noble metal (Silver, Ag)/SiO_2_ nanofibrous mats by combining electrospinning and controlled silica coatings for the detection of Pb^2+^ [99]. 

### 2.3. Detection of Cadmium Ions

**Figure 11:**
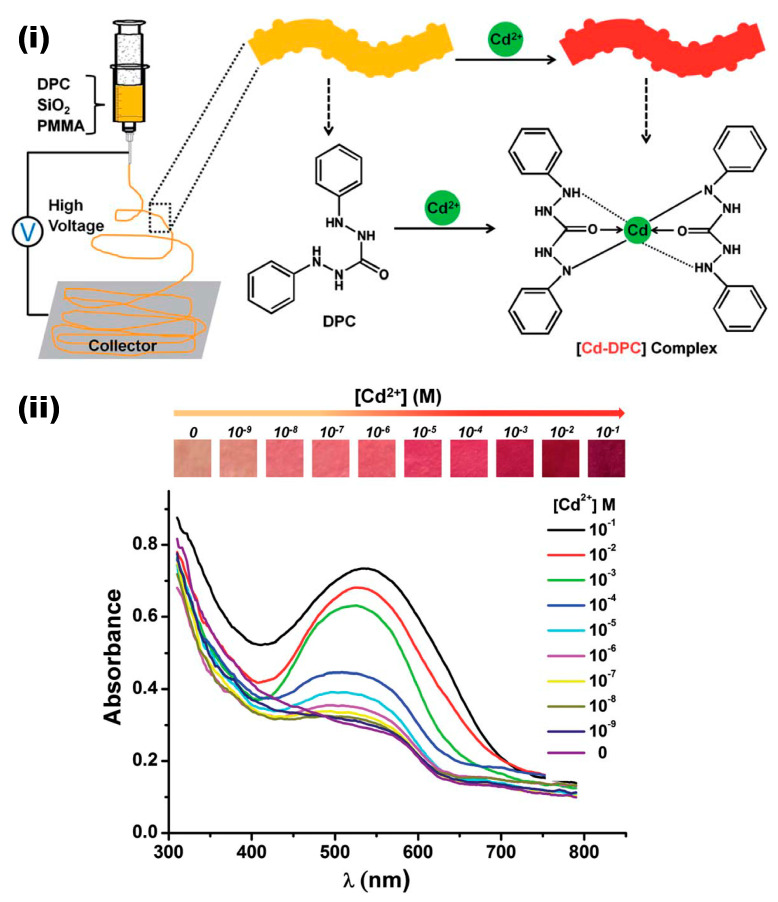
(**i**) Chemical and schematic illustration of the preparation of SiO_2_ nanoparticles and Cd-diphenylcarbazide (DPC)-doped poly(methyl methacrylate) (PMMA) electrospun fibrous film for Cd^2+^ colorimetric detection. (**ii**) Color responses (top optical images) and solid UV-vis absorbance spectra (bottom) of the SiO_2_ and DPC-doped fibrous films after the addition of various concentrations of Cd^2+^. Reprinted with permission from [101], 2014 © The Royal Society of Chemistry.

Cadmium (Cd^2+^) is another important metal pollutant of the environment that causes cancer in the prostate, lungs, pancreas, and kidney, and it also causes system toxicity in the organs of the urinary, skeletal, respiratory, cardiovascular, central, and peripheral nervous systems [100]. Therefore, the detection of such a toxic metal ion is another important area of interest. In this context, very few efforts have been made on the electrospun nanofibers-based colorimetric detection of Cd^2+^. Yao and co-workers adopted the concepts of forming a red color through the Cd-diphenylcarbazide (DPC) complex; the colorimetric detection of Cd^2+^ has been performed through the doping of SiO_2_ nanoparticles and DPC in polymethylmethacrylate (PMMA) electrospun fibers [101]. The chemical and schematic representation of the preparation process and their sensing performances are presented in Figure 11. Briefly, the PMMA, DPC, and SiO_2_ nanoparticles have been dissolved in DMF to prepare the precursor solution and further electrospun to obtain the colorimetric nanofibrous sensor. The obtained sensor was exposed to a range of Cd^2+^ concentrations for a time period of 2 min, and their sensing responses have been recorded. The color response of the nanofibers doped with SiO_2_ nanoparticles and DPC was greater than that of the nanofibers only doped with DPC, since the addition of SiO_2_ nanoparticles was found to increase the roughness and surface area, which helped to improve the sensing behavior of the nanofibers. The sensing results indicated that the sensor membrane turning to a red-pink color following exposure to Cd^2+^ depends on the respective concentration, as can be seen from Figure 11ii. The change in the spectral absorbance revealed that the strong Cd–DPC complex formation increased the absorbance intensity at 523 nm as the concentration increases with the detection limit of 1 × 10^−8^ M. Further, the selectivity experiments revealed an excellent response toward Cd^2+^; however, Cu^2+^ was found to have significant interference, which was further improved by adding the thiourea as a copper masking agent, resulting in improved selectivity [101]. In a similar effort, a PAN/Ag/SiO_2_ nanofibrous film has been explored in Cd^2+^ detection [99].

### 2.4. Detection of Chromium Ions

Chromium (Cr) is a naturally occurring element that exists in a variety of oxidation states ranging from Cr^2+^ to Cr^4+^. Trivalent Cr (Cr^3+^) is an essential element that plays a critical role in the metabolism of protein, glucose, and fat. However, higher-level exposures of Cr^3+^ tend to show severe respiratory, cardiovascular, gastrointestinal, hematological, hepatic, renal, endocrine, and ocular effects [102,103]. In this aspect, the electrospun nanofibers-based colorimetric detection of Cr^3+^ has also been progressed to some extent. In one specific example, Wang et al. prepared 1,4-dihydroxyanthraquinone (1,4-DHAQ) fluorophore-doped cellulose (CL) microporous nanofibers through the electrospinning and deacetylation processes for the purpose of detecting Cu^2+^ in which the fluorescence has been decreased upon exposure to Cu^2+^ [76]. Interestingly, the fluorescence of the quenched membrane has been recovered by the treatment of Cr^3+^ that led to the fabrication of 1,4-DHAQ and Cu^2+^ codoped cellulose ((1,4-DHAQ)-Cu^2+^@CL) nanofiber for the detection of Cr^3+^. The sensing studies revealed that the treatment of Cr^3+^ at the concentrations ranging from 3 to 15 nM resulted in a linear increased fluorescence peak at 424 nm. The sensing mechanism is attributed to the reversible characteristics of the phenolate formed between Cu^2+^ and 1,4-DHAQ. The prepared sensor membrane showed excellent selectivity toward Cr^3+^ against a variety of common interfering cations. In another similar study, carbon nanotubes (CNTs) have been decorated on the 1,4-DHAQ and Cu^2+^ codoped CL nanofiber ((1,4-DHAQ)-Cu^2+^ CNTs@CL) that significantly improved the surface area per unit mass ratio and metal ion sorption capacity, which helped improve the sensing performances based on the sensing mechanism mentioned in the previous investigation. Furthermore, sensing experiments were carried out using the Cr^3+^ polluted lake water to demonstrate their potential practical applications in environmental monitoring [88].

### 2.5. Detection of Mercury Ions

**Figure 12:**
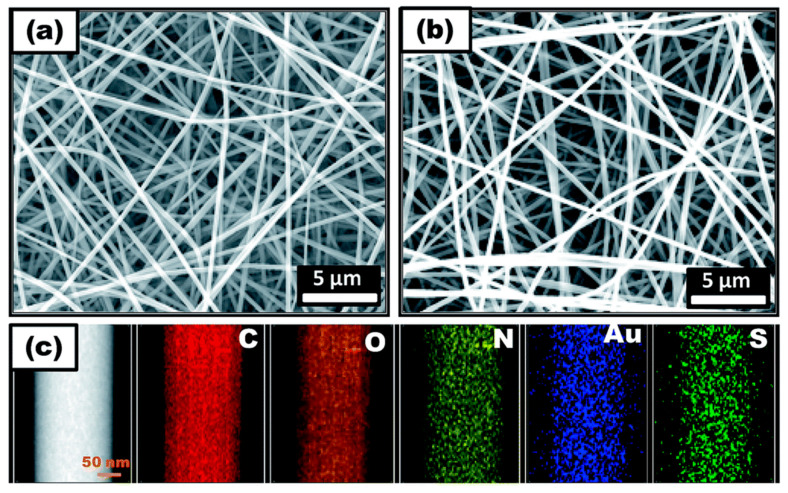
SEM images of the polyvinyl alcohol (PVA) nanofibers (**a**) and gold nanocluster (4 wt %)-embedded PVA nanofibers (**b**). (**c**) HAADF-STEM (high-angle annular dark-field scanning transmission electron microscopy) image and mapping of the elements C, O, N, S and Au present in the AuNC*NF. Reprinted with permission from [107], 2014 © The Royal Society of Chemistry.

Mercury (Hg^2+^) is considered as one of the highly toxic global pollutants posing severe health risks to humans and the ecosystem. Although mercury occurs naturally in heavy metal-rich geologic deposits and coal, the release of mercury into the environment is predominantly caused by rapid industrialization and anthropogenic processes [104,105]. The mercury contamination in the water is a significant concern due to its high toxicity, persistence, and bioaccumulation in the food chain, which can cause severe health effects [106]. The recent past has witnessed a number of pioneer reports focused on the use of electrospun nanofibers and enabled advances in the use of electrospun nanofibers in cutting-edge sensor design. Table 3 summarizes the corresponding studies proceeded for the colorimetric detection of Hg^2+^ by employing different polymeric materials, functional molecules, and approaches along with their performances [86,99,107,108,109,110,111,112,113,114,115,116,117,118,119,120,121]. For instance, Senthamizhan and co-workers reported that a luminescent gold nanoclusters (AuNC) integrated electrospun polyvinyl alcohol (PVA) nanofibrous membrane (NFM), termed AuNC*NFM, has led to the visual colorimetric detection Hg^2+^ in water [107]. The several important issues for fabricating the flexible polymeric nanofibrous membrane composed of AuNC including aggregation, fluorescence quenching, and stability against time and temperature are successfully addressed, and the resultant composite nanofibers have retained the original red fluorescence of AuNC under UV light, which was considered to be one the main criteria for the preparation of a visual colorimetric sensor. The morphology investigation of nanofibers before and after incorporating AuNC represent a defect-free morphology, and the observed surface roughness compared to bare PVA, underlining the partial exposure of the gold nanoclusters on the surface of nanofibers, as shown in Figure 12a,b. In addition, the elemental mapping of a gold nanocluster-incorporated single PVA nanofiber revealed the homogenous distribution of gold nanoclusters along the nanofiber (Figure 12c). The outcome of this has resulted in the purity and homogeneity of the color, which is considered to be the principal factor for the colorimetric sensing properties. Concretely, AuNC*NFM offers great stability over a period of six months at room temperature, and in a parallel way, the fluorescence ability of AuNC*NFM was stable up to a temperature of 100 °C, which implies that temperature does not have a significant effect on the sensing performance in real-time outdoor application. Further, the water stability of AuNC*NFM has been accomplished by cross-linking with glutaraldehyde vapor.

**Figure 13:**
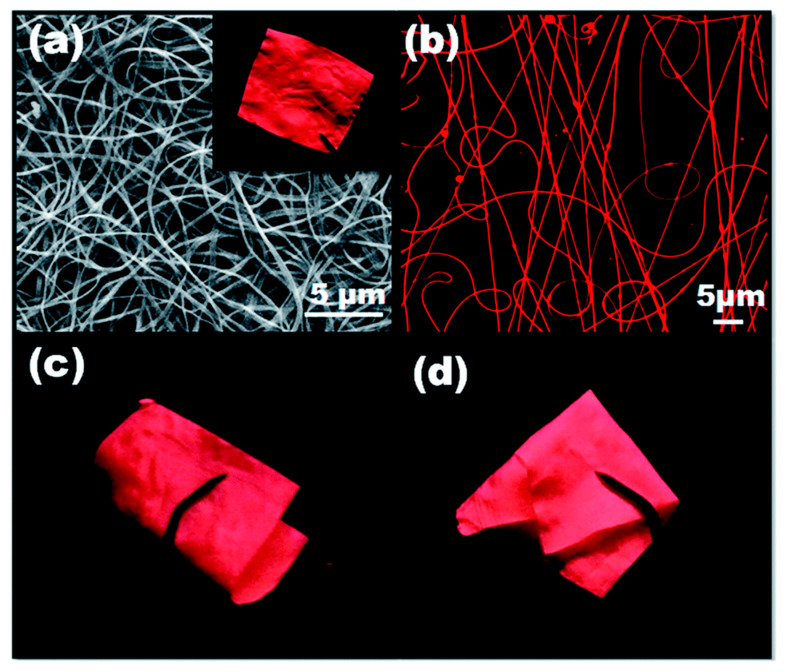
(**a**) SEM image of the cross-linked luminescent gold nanoclusters (AuNC) integrated electrospun polyvinyl alcohol (PVA) nanofibrous membrane (NFM) (AuNC*NFM). The inset shows a photograph taken under UV light. (**b**) confocal laser scanning microscopy (CLSM) image of the AuNC*NF. (**c**,**d**) Flexible nature of the nanofibrous membrane. Reprinted with permission from [107], 2014 © The Royal Society of Chemistry.

Most importantly, the morphology, emission characteristics, and flexibility of the nanofibers are kept unchanged, as represented in Figure 13. In the sensor design, the incorporated nanocluster should not be leached to water as it causes environment pollution. Thereby, the durability of the AuNC*NFM has been tested against water for 24 h, and the result has proven its durability.

**Figure 14:**
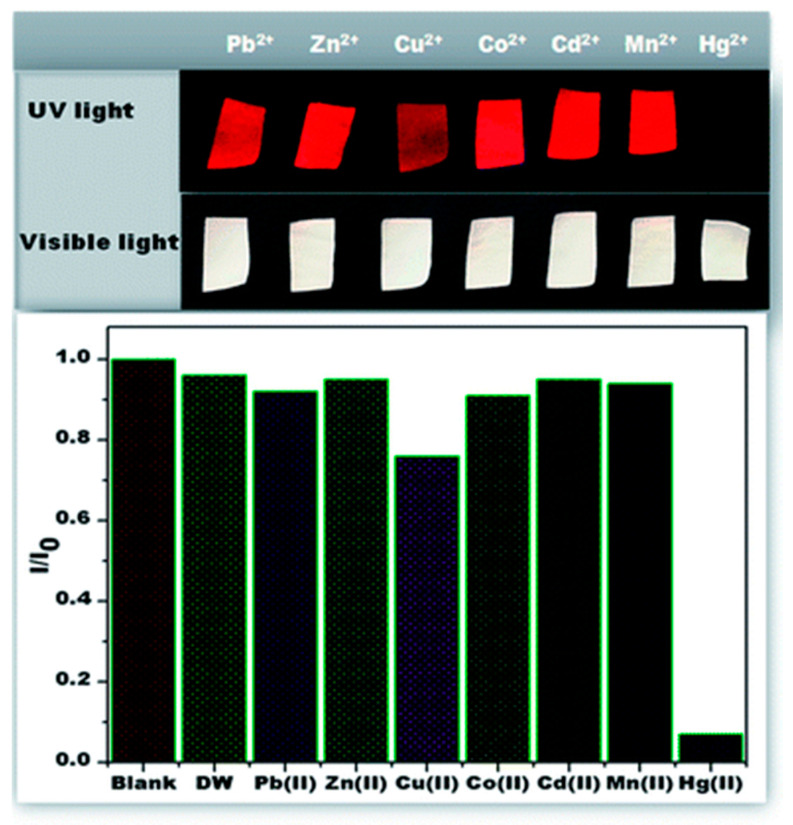
Sensing performance of AuNC*NFM upon exposure to different metal ions in water. The concentration of all metal ions was fixed at 10 ppm. Photographs were taken under UV and white light. Reprinted with permission from [107], 2014 © The Royal Society of Chemistry.

The sensing capability of AuNC*NFM for the detection of mercury ions in an aqueous solution has been tested in various approaches using (i) fluorescence spectra, (ii) visual colorimetric response through contact mode, and (iii) CLSM-based analysis. In an effort to understand the sensing performance, the AuNC*NFM has been immersed in water, and this has resulted in significant changes in the emission behavior viewed through the naked eye. The distinguishable color change has been observed from red to blue upon interaction with mercury, and the detection limit with the naked eye is up to 50 ppb. However, the changes were not visible below the defined concentration. In another approach that used a CLSM method, the detection limit was extended up to 1 ppb, and variations in the emission intensity were observed within 2 min. The important goal in the sensor design is accomplishing selective sensing ability; thereby, the selective sensing performance of the AuNC*NFM has been investigated by its response to other toxic metal ions (Pb^2+^, Mn^2+^, Cu^2+^, Ni^2+^, Zn^2+^, Cd^2+^) in water. The observed results implied that there were no significant changes in the emission, whereas slight quenching occurs following the interaction with Cu^2+^ ions at higher concentration (Figure 14) [107].

**Figure 15:**
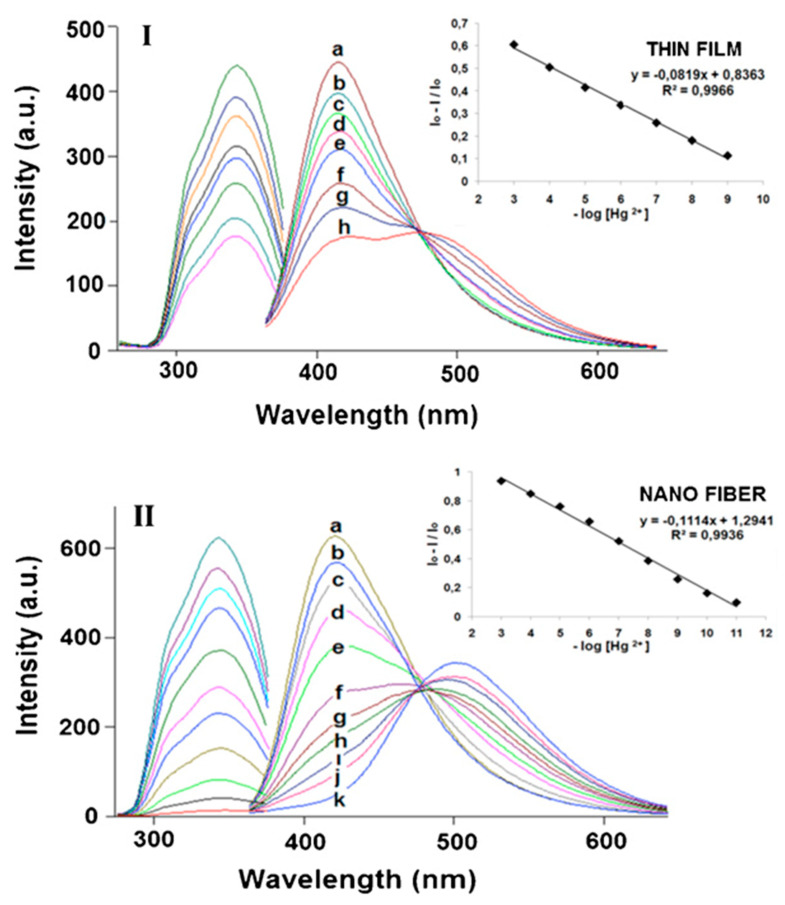
(**I**) Fluorescence response of the EC-based thin film to Hg(II) ions at pH 5.0. (a) Hg-free buffer, (b) 10^−9^ M Hg(II), (c) 10^−8^ M, (d) 10^−7^ M, (e) 10^−6^ M, (f) 10^−5^ M, (g) 10^−4^ M, and (h) 10^−3^ M Hg(II). Inset: Calibration plot for the concentration range of 10^−9^–10^−3^ M Hg(II). (**II**) Response of the EC-based nanofibers to Hg(II) ions at pH 5.0. (a) Hg-free buffer, (b) 10−11M Hg(II), (c) 10−^10^ M, (d) 10^−9^ M, (e) 10^−8^ M, (f) 10^−7^ M, (g) 10^−6^ M, (h) 10^−5^ M, (i) 10^−4^ M, (j) 10^−3^ M, and (k) 10^−2^ M Hg(II), inset: linearized calibration plot for the concentration range of 10^−11^–10^−3^ M Hg(II). Reprinted with permission from [119], 2013 © Elsevier.

In the same field, Ongun et al. fabricated the sensing probe apt for the detection of Hg(II) ions by using a luminescent carbozole derivative dye, 2-(9-methyl-9H-carbazol-3-yl)-5-(pyridin-4-yl)-1,3,4-oxadiazole (ODC-3) [119]. Consequently, the dye-encapsulated ethyl cellulose (EC) nanofibrous matrix has been prepared using the electrospinning approach and the sensing performance toward Hg(II) ions was compared with thin films that were prepared using the same precursor used for the electrospinning. As a result, electrospun fibers exhibited high sensitivity and reactivity as compared to thin films, as shown in Figure 15. A closer examination of the observed results perceived that the spectral response to Hg(II) was similar for both thin film and electrospun materials. Upon the addition of Hg(II) ions, the emission intensity observed at 418 nm was decreased, whereas the intensity was observed at 505 nm. The noticed dual fluorescence properties allowed them to be classified as ratiometric sensors. In addition to changes in the emission spectra, a distinguishable color change has been noticed from colorless to yellow. The detection limits of the sensor were calculated to be 1.70 × 10^−15^ M and 1.90 × 10^−13^ M for electrospun nanofiber and thin films, respectively. The findings in this work proved the advantages of the nanomaterial design in sensor development for the enhanced sensitivity. The electrospun nanofibrous membrane shows an excellent Stern–Volmer relationship in a linear range of response from 1 × 10^−11^ M to 1 × 10^−6^ M [119].

Despite the potential advantages of the direct encapsulation approach described above, there are some significant changes that need to be addressed further in order to improve the sensing performance. A crucial point in the sensor design is the limited accessible sensing unit inside the fibrous matrix. In order to overcome this issue, as a pioneer work, Senthamizhan et al. decorated the gold nanoclusters on the surface of the electrospun polycaprolactone (PCL) nanofibers for the detection of mercury ions [117]. A simple and effective dip-coating approach has been adopted to coat the gold nanoclusters on the surface of the PCL fibers, and the number of considerations influence the anchored gold nanoclusters for sensing Hg(II) in water have been studied. First and foremost, the coating density of the gold nanocluster on the nanofiber surface has been optimized by controlling the processing time and their associated emission properties. As a result, a patch-like structure was observed on the PCL nanofiber surface when the incubation time in the gold nanocluster increased from 12 to 24 h. A detailed investigation on the emission spectra concluded that the saturation time for attaining the optimal gold nanocluster on the surface of the PCL nanofiber was ≥2 h. In addition, the non-specifically adsorbed excess ligand on the nanofiber surface has been removed effectively by a ligand extraction procedure, as it was found to suppress the binding ability of gold nanocluster with analytes, and more importantly, it is expected to interrupt the selective sensing performance.

**Figure 16:**
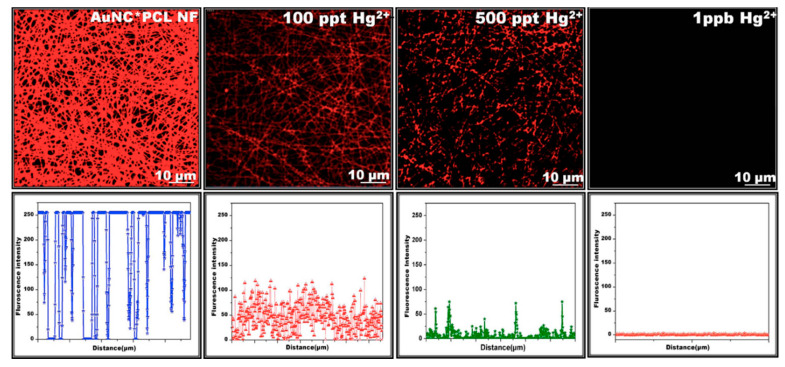
Sensing performance of AuNC*PCL-NF toward Hg^2+^. CLSM images of AuNC*PCL-NF before and after exposure to various concentration of Hg^2+^ and their line scanning profiles, which were recorded across each spot. The gradual decreases of fluorescence in the AuNC*PCL-NF upon increasing the concentration of Hg^2+^ were noticed. The formation of fiber junctions during the electrospinning process limits the diffusion of mercury ions between these junctions; thereby, a fewer number of brighter spots are visually seen in 500 ppt Hg^2+^-treated AuNC*PCL-NF. Reprinted from [117], 2015, Springer Nature.

Overall, the detailed investigations indicated that the optimal time for coating was 3 h followed 30 min of washing. In addition, it has been confirmed that the gold nanoclusters were not detached from the surface of the nanofiber even after dipping in water for a prolonged period of time (12 to 24 h). Thus, the most favorable colorimetric sensing probe (AuNC*PCL-NF) was attained, and the sensing performance of AuNC*PCL-NF for the detection of Hg^2+^ has been performed by dipping them in Hg^2+^ contaminant water for about 10 min; then, their corresponding fluorescence spectra have been observed. The fluorescence intensity of AuNC*PCL-NF is gradually decreased upon increasing the concentrations of Hg^2+^ and remarkably at higher concentration (ppm), the strong interaction between the gold and mercury has led to the blue shift in the spectra. The Hg^2+^ sensing performance using CLSM is illustrated in Figure 16.

**Figure 17:**
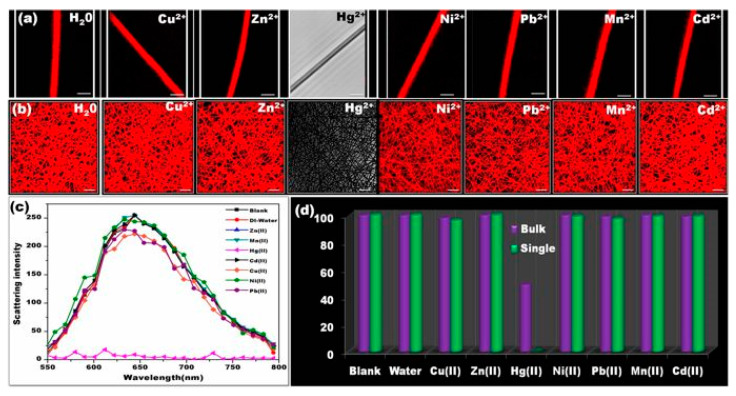
Selective sensing performance of an AuNC*PCL-SNF and an AuNC*PCL-NF mat. CLSM image presents the fluorescence response of an AuNC*PCL-SNF (**a**) and an AuNC*PCL-NF mat (**b**) to various metal ions (indicated in each image) at a concentration of 10 ppm. (Scale bar: Figure a-2 μm and b-5 μm) (Note: For Hg^2+^ only, differential interference contrast (DIC) images are given, since fluorescence is completely quenched and the CLSM image become fully black). The H_2_O-treated AuNC*PCL-NF shows their stability as well proves that the observed decreased fluorescence upon the addition of metal ions is not because of solvent. (**c**) Variation in the emission spectra of different metal ions treated with AuNC*PCL-SNF. (**d**) Bar diagram illustrating the relative variation in the fluorescence intensity of the single NF and nanofibrous mat. Reprinted from [117], 2015, Springer Nature. PCL: polycaprolactone, SNF: single nanofiber.

To extend the applicability of this approach, a single nanofiber (SNF)-based sensor approach has been adopted. The observed results have strongly suggested that the sensing response was uniform throughout the nanofiber surface, which long-established that each single nanofiber can competently act as an independent sensor, offering an opportunity to devise a low-cost sensor. However, despite numerous developments, a fast response time is considered to be an important factor in estimating the practical application of a sensor. The unique properties of AuNC*PCL-NF makes them suitable as fast sensing probes in this field. Incredibly, the single nanofiber-based sensor shows a faster response time than bulk nanofiber ensembles. The prepared AuNC*PCL-NF showed an excellent selective sensing performance for Hg^2+^ over other competent metal ions such as Cu^2+^, Ni^2+^, Mn^2+^, Zn^2+^, Cd^2+^ and Pb^2+^ present in the water, as depicted in Figure 17. The accomplished specific selectivity toward Hg^2+^ over competent Cu^2+^ ions has been successfully obtained in the absence of an excess amount of ligand on the nanofiber surface [117].

**Figure 18:**
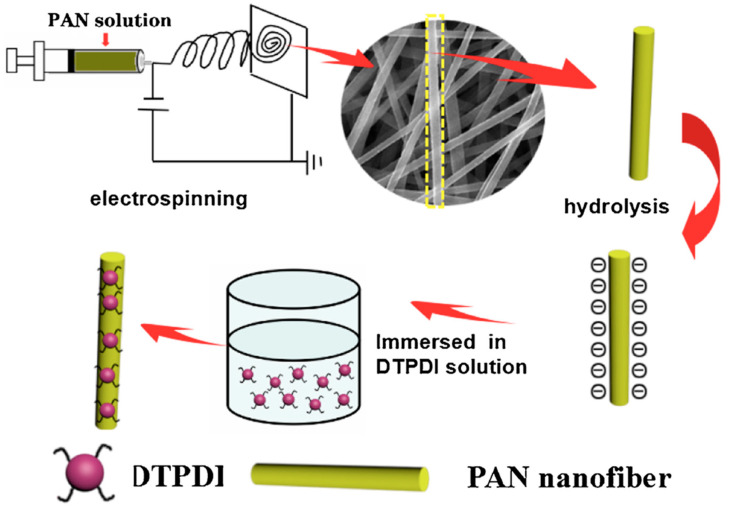
Schematic illustration for the fabrication of FNFM (fluorescent nanofibrous membrane (FNFM) for the detection of mercuric ion (II) with high sensitivity and selectivity). Reprinted with permission from [109], 2017 © Elsevier.

The progress in the surface functionalization approach has evoked a growing interest in the development of an advanced sensor device. Correspondingly, in another study, Ma et al. developed a fluorescent chemosensor for the detection of Hg^2+^ [109]. The sensor has been prepared by decorating the dithioacetal-modified perylenediimide (DTPDI) on the surface of polyacrylonitrile (PAN) nanofibers, as illustrated in Figure 18. The fabrication procedure for a fluorescent nanofibrous membrane (FNFM) was composed of three steps as follows: (i) PAN nanofibers were fabricated using the electrospinning method; (ii) the surface of the PAN nanofibers was treated with sodium hydroxide solution to create the carboxyl groups on their surface; (iii) and as a final step, the FNFM was prepared by dipping the hydrolyzed PAN nanofibers in DTPDI solution. The successful decoration of a fluorescent probe has been achieved via an electrostatic interaction between positively charged DTPDI and negatively charged nanofibers. The sensing performance of FNFM revealed an excellent sensitivity for the detection of Hg^2+^ with a detection limit of 1 ppb. Besides, the FNFM showed an excellent selectivity for Hg^2+^ over other competent metal ions including K^+^, Na^+^, Mg^2+^, Ca^2+^, Cu^2+^, Zn^2+^, Fe^2+^, Fe^3+^ and Cr^3+^ ions. The responsible sensing mechanism of FNFM to Hg^2+^ can be expressed as special sulfur-mercury affinity. Perhaps the greatest challenge in the sensing field is achieving the reversibility, as the durability of the sensor significantly reduces the cost of detection and environmental issues. In this view, the prepared FNFM was reversible and can be repeatedly used 7 times [109].

In another effectual approach, electrospun fluorescent polymers offer a reliable option for the advanced sensing field due to their inherent emission characteristics, easy fabrication procedure into fibers, and limited environmental issues, as this material does not require any additional coating. The intensive research reports have already well proven that electrospun nanofibers can offer an enhanced sensitivity as compared to thin films. In this regard, the conceptual underpinning has been initiated with a model study published by Wang et al. [108]. The fluorescent electrospun propoly(acrylic acid)−poly(pyrene methanol) (PAA−PM) cross-linked with a polyurethane polymer nanofibrous membrane was used as an optical sensor for metal ions and 2,4-dinitrotoluene (DNT) detection. The fluorescent PAA−PM has been produced by the covalent functionalization of pyrene methanol (PM) into poly(acrylic acid) (PAA). From the morphological evaluation, it was discerned that the randomly oriented nanofibers were uniformly coated on the substrate. The developed optical sensors displayed an excellent sensing performance toward electron-deficient metal cations (Hg^2+^ and Fe^3+^) and nitro aromatic compounds (2,4-dinitrotoluene (DNT) and trinitrotoluene (TNT)). The subsequent discussion emphasizes that the quenching performance followed the Stern−Volmer relationship in the quencher concentrations range of 10^−6^ to 10^−7^ mol/L. The calculated Stern−Volmer constants (*K*_sv_) of the optical sensors were 1.1 × 10^6^ (M^−1^), 8.9 × 10^5^ (M^−1^), and 9.8 × 10^5^ (M^−1^) for Fe^3+^, Hg^2+^, and DNT, respectively [108].

In the recent past, there has been heightened interest in the application of nanostructured forms of polyaniline (PANI), which is a well-known conjugated polymer in the field of chemical sensors. The oxidation states of the PANI leucoemeraldine base (fully reduced, PANI-LB), emeraldine base (half oxidized, PANI-EB), and pernigraniline base (fully oxidized, PANI-PB) have highly depended on the ratio of reduced benzenoid amine to oxidized quinoid imine. Attractively, the presence of substantial amounts of amine and imine functional groups of PANI are anticipated to have a unique interaction or complexation with Hg^2+^. For example, Si et al. developed a radically different type of PANI-based immobilized sensor for the naked-eye colorimetric detection of Hg^2+^ in water [120]. Three parameters have been considered as an important criterion: (I) the selective sensing response to Hg^2+^ in aqueous solution, (II) the homogeneous immobilization of PANI on the substrate, and (III) the sensing membrane should be hydrophilic, porous, and highly stable in water. As a first requirement, the PANI-LB has been selected as a sensing probe due to its ability to “turn-on” and “color-change” upon interaction with Hg^2+^.

**Figure 19:**
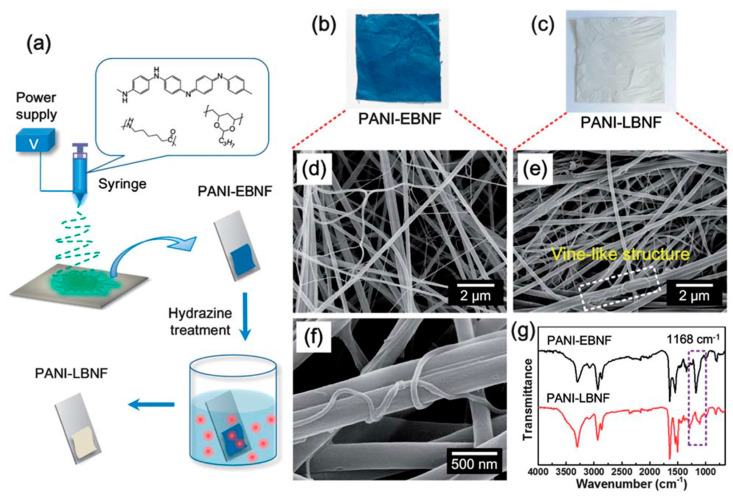
(**a**) Schematic illustration of the preparation of polyaniline leucoemeraldine base nanofibrous (PANI-LBNF) sensing membranes by the combination of blending electrospinning and hydrazine treatment. Optical images of (**b**) as-spun polyaniline emeraldine base nanofibrous (PANI-EBNF) and (**c**) PANI-LBNF membranes. FE-SEM images of (**d**) PANI-EBNF and (**e**) PANI-LBNF membranes. (**f**) High-magnification FE-SEM image of PANI-LBNF showing the vine-like hierarchical structure. (**g**) Fourier-transform infrared spectroscopy (FT-IR)FT-IR spectra of PANI-EBNF and PANI-LBNF samples. Reprinted with permission from [120], 2014 © The Royal Society of Chemistry.

To briefly resume this point, the selective sensing process involved a series of redox and doping reactions. The analysis of the results indicated that the involved reactions strongly depend on the concentration of Hg^2+^ and reaction times. In a parallel way, the electrospinning method in combination with an in situ hydrazine reduction treatment has been proven to be a promising approach to produce the homogeneous PANI-LB immobilized membranes with high porosity, hydrophilicity, and stability. The schematic representation of the preparation procedure for PANI-LBNF sensing membranes has been depicted in Figure 19. The multicomponent nanofibrous membrane (PANI-EBNF) composed of PANI-EB, PVB, and PA-6 has been prepared in which polyvinylbutyral (PVB) and polyamide-6 (PA-6) were selected as template material due to their good spinnability, strength, and good miscibility with PANI. Subsequently, after removing the residual solvent, PANI-EBNF membranes were fixed successfully on the glass surface. Then, a reduced PANI-LB sensing probe has been prepared through the in situ reduction of PANI-EBNF at 50 °C in a 40 wt % hydrazine aqueous solution.

**Figure 20:**
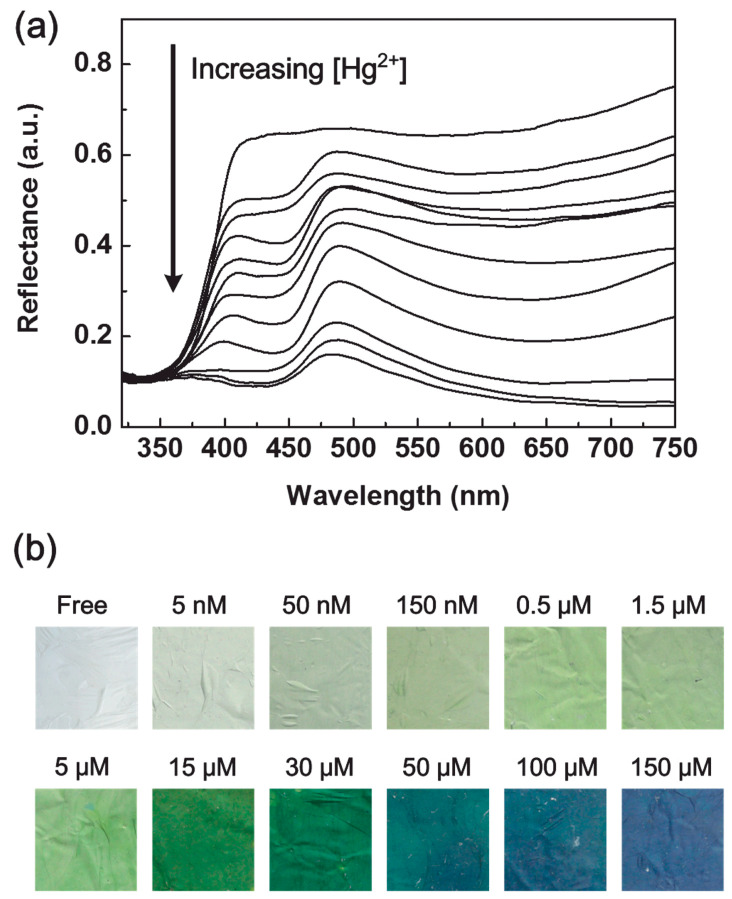
(**a**) Reflectance spectra and (**b**) optical colorimetric responses of the PANI-LBNF sensor strips after incubation for 20 min in Hg^2+^ aqueous solutions with concentrations of 0, 5 nM, 50 nM, 150 nM, 0.5 μM, 1.5 μM, 5 μM, 15 μM, 30 μM, 50 μM, 100 μM, and 150 μM. Reprinted with permission from [120], 2014 © The Royal Society of Chemistry.

The sensing membrane was cut into small square sensor strips (15 mm × 15 mm) and then immersed into the aqueous solution of Hg^2+^ for 20 min under gentle stirring. Depending on the concentrations of Hg^2+^, the reflectance intensity of PANI-LBNF sensor membrane has been decreased as shown in Figure 20a, denoting incredible variations in the optical feature. In addition to this, determining the color change was considered as another important factor. The corresponding results have shown the distinguishable color change from white to yellow/green, green, and blue upon the increasing Hg^2+^ concentration, which could be visibly recognized with the naked eye, as presented in Figure 20b.

The prepared PANI-LBNF membrane exhibited excellent selectivity toward Hg^2+^ ions over common cations (5 μM) including Na^+^, K^+^, Mg^2+^, Ca^2+^, Ba^2+^, Pb^2+^, Mn^2+^, Co^2+^, Ni^2+^, Zn^2+^, Cd^2+^, and Al^3+^ whereas slight changes were induced by Fe^3+^ and Cu^2+^. The perceived selective binding can be explained by two factors: (I) the electrode potential of Hg^2+^ is comparable to the oxidation potential of PANI-LB, which caused the “turn-on” signals; and (II) the formation of Hg^2+^–PANI complexes via a two-step redox doping reaction between Hg^2+^ and PANI. In addition to the selective sensing competence, determining the reversibility of the PANI-LBNF sensor is essential and meaningful from an economic point of view. In general, it remains a great challenge to preserve the bare fluorescence after a number of sensing and regeneration cycles. The reversibility of the PANI-LBNF sensor has been done by hydrazine treatment procedures and even after 5 regeneration/reuse cycles, the fluorescence of PANI-LBNF remains unaffected. The high reversibility of the PANI-LBNF sensor strip is mainly attributed to the great immobilization of PANI-LBNF probes in electrospun nanofibrous membranes and the robust stability of the interconnected hierarchical fibrous structure [120].

**Figure 21:**
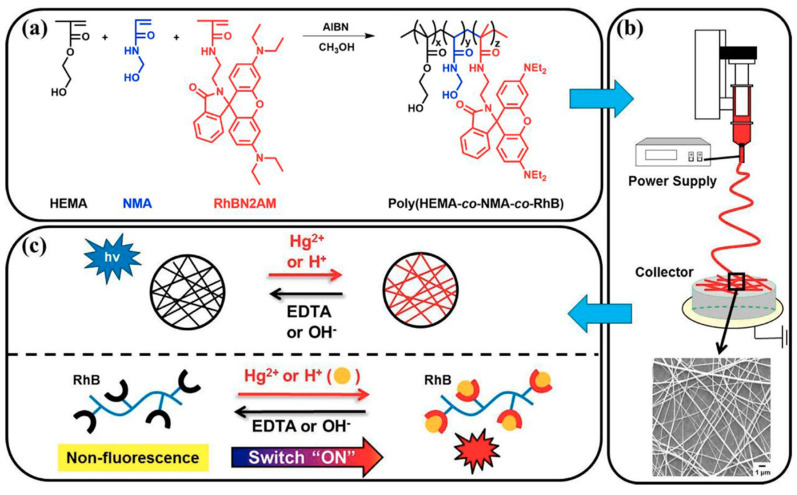
Design of fluorescent electrospun nanofibers with multifunctional detection. Schematic representation of (**a**) poly(2-hydroxyethyl methacrylate-*co*-N-methylolacrylamide-*co*-RhBN2AM) (poly(HEMA-*co*-NMA-*co*-RhBN2AM)] copolymers synthesis, (**b**) fabrication of electrospun nanofibers and (**c**) their Hg^2+^ sensing mechanism. Reprinted with permission from [111], 2017 © Elsevier.

Chen and co-workers developed another reliable method for the sensing of mercury ions (Hg^2+^) and pH using electrospun fluorescent chemosensory filter membranes [111]. The single-capillary spinneret approach has been adopted to prepare the nanofibers from poly(2-hydroxyethyl methacrylate*-co-N*-methylolacrylamide*-co-*rhodamine derivative) (poly(HEMA*-co-*NMA*-co-*RhBN2AM)). The schematic representation of the preparation procedure is illustrated in Figure 21. The prepared electrospun nanofibers essentially consist of three functional segments: hydrophilic hydrogel material (PHEMA), chemical cross-linking segment (PNMA), and fluorescent probe (RhBN2AM) sensitive to Hg^2+^and pH. The result of the sensing experiments showed that the emission of RhBN2AM within electrospun nanofibers was highly selective to Hg^2+^ and pH dependent.

In more detail, the spirocyclic form of RhBN2AM is colorless and nonfluorescent in neutral or alkaline media or an aqueous solution without Hg^2+^, and it exhibits strong fluorescent pink emission (ring-opened acyclic form) in acidic media or an aqueous solution with Hg^2+^. The subsequent discussion emphasizes that the on/off switching emission characteristics of the electrospun fluorescent chemosensory filter membrane can be certainly modified by adjusting either the concentration of Hg^2+^ or pH value. To determine the ability of selectivity, different metal ions such as Co^2+^, Cu^2+^, Ni^2+^, Mg^2+^, Zn^2+^, Cd^2+^, Pb^2+^, Na^+^, and K^+^ were tested. As anticipated, only Hg^2+^ displayed significant on/off switchable fluorescence emission. More importantly, the Hg^2+^-induced fluorescence emission was successfully recovered to the original value obtained in the absence of Hg^2+^ by the addition of ethylenediaminetetraacetic acid (EDTA). From a practical perspective, it is also worth highlighting that the chemosensory membrane exhibited high reversibility [111].

### 2.6. Detection of Nickel Ions

Nickel (Ni^2+^) is also a heavy metal that is present in the environment at much lower concentration, and it is considered an essential element for biological systems. Nevertheless, the significant discharge of Ni^2+^ from several industries including electroplating, power plants, nickel-cadmium battery, rubber, and plastic industries leads to environmental pollution, thus causing Ni^2+^ induced carcinogenicity, genotoxicity, immunotoxicity and effects in other vital organs including lung [122,123,124]. An early study used a dimethylglyoxime (DMG)/poly(caprolactone) (PCL) blend for the fabrication of optical sensors through electrospinning [125]. Firstly, the DMG has been dissolved with PCL in a mixture of DMF and dichloromethane (DCM) (50/50, *v*/*v*); then, the precursor solution was subjected to the electrospunning process. Based on the detailed characterization, the optimum mass ratio between the DMG and PCL has been found to be 20:80, as the produced nanofibers had the smallest diameter distribution and best spinning characteristics. The sensing experiments were performed by placing 3 × 3 cm^2^ electrospun fiber mats in the Ni^2+^ solution for a period of 10 min, and then the color change was observed. The corresponding results showed a significant color change due to the formation of a red-pink complex of Ni^2+^and DMG with a detection limit of 1 ppm [125]. In another similar example, DMG and PCL composite nanofibers have been collected on a glass slide and further impregnated with a polyvinyl alcohol (PVA) to make them transparent prior to spectroscopy analysis [126]. The sensing behavior of the membrane has been observed from the formation of a bright red Ni(DMG)_2_ complex attributed to the interaction between the Ni^2+^ and DMG from electrospun nanofibers. The designed nanofibrous sensor showed an improved sensing performance with a limit of detection of 2 µg mL^−1^. The effect of co-existing existing ions has been studied, and the results indicated good selectivity toward Ni^2+^; however, Cu^2+^ had shown significant interference at higher concentrations. Furthermore, the Ni^2+^ sensing performance also has been demonstrated using laboratory waste water, tap water, and electroplating nickel pool [126]. Adewuyi et al. made an effort in the determination of Ni^2+^ through the electrospinning of covalently functionalized pyridylazo-2-naphthol-poly(acrylic acid) polymer [127]. Initially, fluorescent polymer has been synthesized by esterification of PAN and poly(acrylic acid) (PAA)in the presence of 1’,10-carbonyldiimidazole (CDI) and 1,8-diazabicyclo[5.4.0]undec-7-ene (DBU) that resulted in fluorescence pyridylazo-2-naphthol-poly(acrylic acid) (PAN-PAA), which has been further electrospun and crosslinked with β-cyclodextrin to fabricate a “turn-off” fluorescence sensor. The sensing experiments have been performed using an optical cell containing a glass slide coated with fluorescent electrospun nanofibers. The results indicated fluorescence quenching behavior upon exposure to Ni^2+^ solution, owing to the formation of a complex between Ni and PAN-PAA. The developed sensor achieved a detection limit of 0.07 ng mL^−1^ and their selectivity experiments resulted in the scarcely fluorescent quenching behavior of other metal ions. The formed complex can be reversible by the treatment of HCl that helped with the evaluation of its reusability nature, and the corresponding findings indicated that the Ni^2+^ was able to recombine with PAN-PAA four times [127]. Another study demonstrated the determination of Ni^2+^ using polyacrylonitrile (PAN)/noble metal (Silver, Ag)/SiO_2_ nanofibrous mats [99].

### 2.7. Detection of Zinc Ions

**Figure 22:**
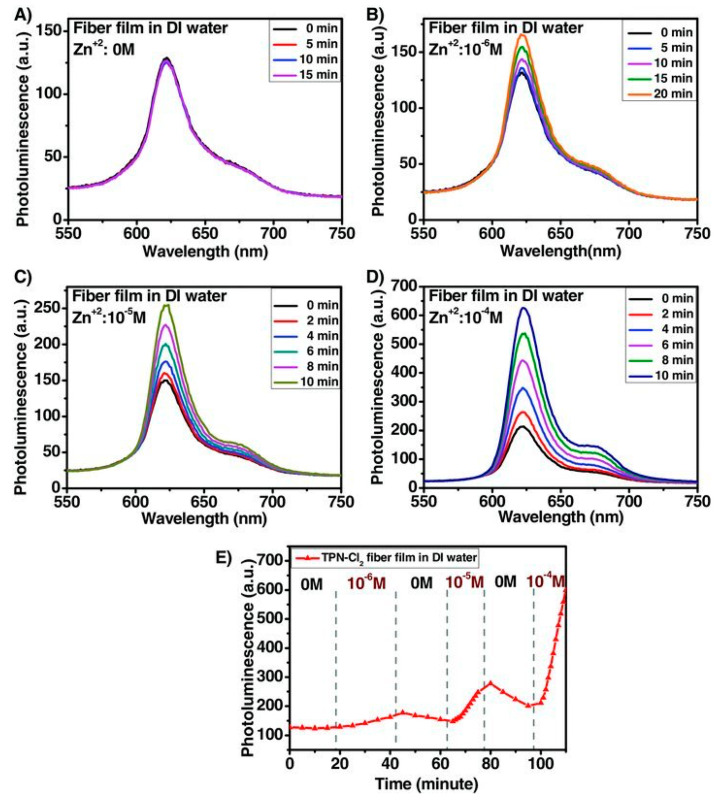
Time dependence of the PL spectra for the fiber film in deionized water (DI water) with zinc ions, measured with a PL spectrometer: (**A**) 0 M zinc ions; (**B**) 10^−6^ M zinc ions; (**C**) 10^−5^ M zinc ions; and (**D**) 10^−4^ M zinc ion. (**E**) The PL spectra in DI water with different concentrations of zinc ions. Reprinted with permission from [130], 2013 © John Wiley and Sons.

Zinc (Zn^2+^) has been apparently used by humans for centuries, and it plays a vibrant role in the human physiology and pathology. Although Zn^2+^ has been considered as an essential and nontoxic element, excessive exposure to Zn^2+^ has a severe impact on the central nervous system by means of nerve cell injury, which causes effects ranging from strokes to Alzheimer’s disease [128,129]. Therefore, the colorimetric determination of Zn^2+^ has also been attracted accountable interest; correspondingly, very few efforts have been directed toward the electrospun nanofiber-based colorimetric detection of Zn^2+^. For example, *meso*-2,6-dichlorophenyltripyrrinone (TPN-Cl_2_), a red fluorescent probe sensitive to Zn^2+^ has been doped into hydrogel polymer poly(2-hydroxyethyl methacrylate) (poly HEMA) and electrospun to obtain nanofibrous film that was further used for the detection of Zn^2+^ in water [130]. A planar film has been also fabricated using similar composition for the purpose of comparing their sensing efficiency. The photoluminescence spectra of the TPN-Cl_2_-doped poly HEMA nanofibrous film showed that the introduction of Zn^2+^ in the concentration range of 0–10^−6^ M demonstrated that the emission peak at 620 nm was responsible for Zn^2+^, which becomes a shoulder, remains responsive, and is attributed to the formation of a complex between Zn and TPN-Cl_2_ (see Figure 22). The sensing performance of the fibrous film has been noted with a detection limit of 10^−6^ M, which is higher than the planar film [130].

Another representative study demonstrated the fabrication of multifunctional electrospun nanofibers using copolymers of poly{2-{2-hydroxyl-4-[5-(acryloxy)hexyloxy]phenyl}benzoxazole}-*co*-(N-isopropylacrylamide)-*co*-(stearyl acrylate)} (poly(HPBO-*co*-NIPAAm-*co*-SA)) through the free radical polymerization process and electrospinning [131]. As an initial process, the 2-(2-hydroxyphenyl)benzoxazole (HPBO) monomer has been synthesized. Later, poly(HPBO-*co*-NIPAAm-*co*-SA has been prepared using the free-radical copolymerization of three monomers, HPBO, NIPAAm, and SA, which have been further used for electrospinning and the drop-casting process. The sensing experiments have been carried through fixing the nanofibrous membrane in the cuvettes followed by exposure to metal ions at the concentration ranging from 10^−10^ to 10^−4^ M for a time period of 5 min. The treatment of Zn^2+^ resulted in an apparent blue shift from 470 to 410 nm with considerable color change to sky-blue from green-blue fluorescence, owing to the interruption of the excited-state intramolecular proton transfer (ESIPT) process due to zinc complexation. The membranes prepared with the monomer at the composition ratio 1:93:6 of HPBO/NIPAAm/SA (**P4**) showed ultrasensitive detection as low as 10^−8^ M. Moreover, the beneficial features of the nanofibers significantly enhanced the sensing performances over the thin films [131]. A similar approach has been used for the fabrication of electrospun nanofibers using binary blends of poly(2-hydroxyethyl methacrylate-*co*-N-methylolacrylamide) (poly(HEMA-*co*-NMA)) and 9,9-dihexylfluorene-2,7-bipyridine (bpy-F-bpy) and further used in Zn^2+^ sensing application [74]. Another study used copolymers of poly{(N-isopropylacrylamide)-*co*-(stearyl acid)-*co*-[9,9-dihexylfluorene-2-bipyridine-7-(4-vinylphenyl)]} (poly(NIPAAm-*co*-SA-*co*-FBPY) for the electrospun nanofibers preparation and used them in the sensing of Zn^2+^, and the corresponding results showed the performance with the detection limit of 10^−5^ [132].

### 2.8. Detection of Iron Ions

Iron (Fe^3+^) is most biologically essential element that contributes to deoxyribonucleic acid (DNA) synthesis, the formation of hemoglobin, enzymatic reaction, oxygen transport, and electron transport. Although iron deficiency leads to several pathological disorders, excess exposure results in free radical formation that causes severe damages in various tissues and also causes neurodegenerative diseases [133,134]. Therefore, the detection of Fe^3+^ in water also holds promising importance in monitoring of Fe^3+^ pollution. Table 4 summarizes the corresponding studies proceeded for the colorimetric detection of Fe^2+^ and Fe^3+^ by employing different polymeric materials, functional molecules, and approaches along with their performances [82,99,108,115,135,136,137,138,139,140,141,142,143,144,145]. To complement, cemented efforts have been directed toward adopting several approaches in the designing of electrospun nanofiber-based colorimetric sensors for the detection of Fe^3+^. A very early study reported by Wang et al. used electrospun nanofibers of fluorescent polymer poly(acrylic acid)-poly(pyrene methanol) (PAA-PM) through electrospinning and thermally cross-linkable polyurethane latex mixture solutions for the detection of Fe^3+^ [108]. The fluorescence of the sensing membrane has been quenched by the Fe^3+^ exposure due to the interaction of an electron-rich PM indicator^(–)^ and electron-deficient quencher^(+)^ [108]. Another investigation used a similar sensing mechanism where hexaphenylsilole (HPS) as a functional probe has been blended with polymethyl methacrylate (PMMA) polymer for the preparation of an electrospun nanofibrous composite membrane [115]. The prepared membrane has been evaluated for its sensing performance toward Fe^3+^, and the results indicated a gradual decrease in the fluorescence intensity attributed to the quenching of the HPS indicator by Fe^3+^ [115].

Saithongdee and co-workers have prepared an electrospun zein membrane containing curcumin through electrospinning fabricated by electrospinning and amide crosslinking by a heat-induced process with citric acid, which has been further employed in optical Fe^3+^ detection [135]. After detailed characterization on their morphology, structural, and optical features, the sensing ability of the membrane has been tested by immersing the membranes in Fe^3+^ for 3 h, and their response has been observed using the naked eye. The observations revealed that at pH 2, the color of the membrane turns from yellow to brown with the lowest detection limit of 0.4 mg/L. Furthermore, the experiments with co-existing metal ions demonstrated excellent selectivity toward Fe^3+^, as no significant color change has been noted with other ions [135]. A similar effort has been made using electrospun nanobelt-shaped curcumin-loaded zein membranes for the detection of Fe^3+^, and their detection limit was determined as 0.3 mg/L [136]. Additional effort has been progressed using the combination of ethyl cellulose (EC) polymer, N’-(4-cyanobenzylidene) isonicotinohydrazide (CBINH) indicator dye, and ionic liquid [1-ethyl- 3-methylimidazolium tetrafluoroborate] [137]. These components have been electrospun to obtain a nanofibrous membrane and thin film. The sensing performance of the prepared membrane has been evaluated, and the results indicated that Fe^3+^ treatment quenched the fluorescence of the membrane owing to the formation of a complex between the dye and Fe^3+^. The sensing performance of the nanofibrous membrane was demonstrated with the limit of detection of 0.07 pM within 30 s, whereas the thin film achieved the lowest detection limit of 6 pM [137]. In continuation to these efforts, electrospun nanofibers of 1,10-phenanthroline (Phen) with polycaprolactam (PA6), 4,4′-fluoresceinoxy bisphthalonitrile (FPN) with polycaprolactone (PCL), and a poly(aspartic acid) (PASP) electrospun nanofiber hydrogel membrane have been fabricated for the detection of Fe^2+^ (detection limit: 1 µg/mL), Fe^3+^ (detection limit: 2.9413 nM), and Fe^3+^ (detection limit: 0.1 mg/L) respectively [82,138,139]. Specifically, the study reported by Zhang et al. demonstrated the fabrication of a PASP electrospun nanofibrous hydrogel membrane (PASP−ENHM) through the polymerization of aspartic acid; then, it was used for the detection of Fe^3+^. The Fe^3+^ sensing of PASP-NH has shown that the reflectance intensity of the sensors at 430 nm has been significantly decreased as the concentration increased, and the visual response demonstrated the white-to-yellow color change with the detection limit of 0.1 mg/L [82]. In another study, the plasmon-enhanced fluorescence (PEF) activity concept was adopted in demonstrating the fabrication of polyacrylonitrile (PAN)/noble metal (Silver, Ag)/SiO_2_ nanofibrous mats by combining electrospinning and controlled silica coatings for the detection of Fe^3+^ [99]. Another representative study showcased the fabrication of an electrospun nanofibers-based Fe^3+^ sensor using poly(methyl methacrylate) (PMMA), poly(vinyl chloride-*co*-vinyl acetate-*co*-vinyl alcohol) (PVC terpolymer), and pyrene [140].

**Figure 23:**
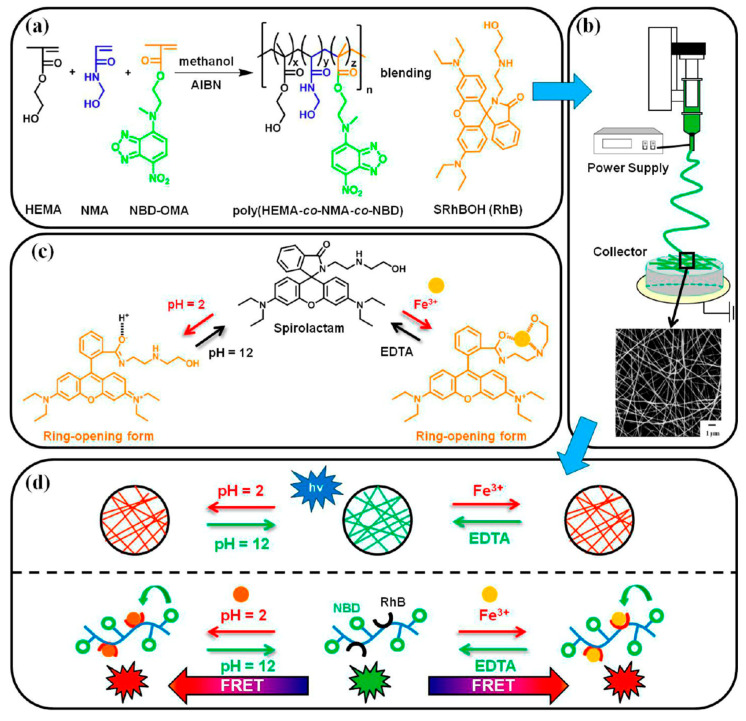
Design of multifunctional sensory ES nanofibers from poly (2-hydroxyethyl methacrylate-*co*-N-methylolacrylamide) and spirolactam rhodamine derivative (poly(HEMA-*co*-NMA-*co*-NBD)/SRhBOH) blends with fluorescence emission in various colors. (**a**) Polymerization and chemical structure of poly(HEMA-co-NMA-co-NBD) and SRhBOH. (**b**) Fabrication of ES nanofibers from the blends. (**c**) The change in the chemical structure of SRhBOH in solutions with Fe^3+^ or ethylenediaminetetraacetic acid (EDTA) or pH 2 or pH 12. (**d**) The effect of fluorescence resonance energy transfer (FRET) on the change of fluorescent emission colors of multienvironment sensing ES nanofibers. Reprinted with permission from [141], 2015 © American Chemical Society.

The incorporation of functional molecules through the polymerization approach and subsequent electrospinning process for obtaining colorimetric sensors for Fe^3+^ detection has also attracted significant interest. In this aspect, Chen and co-workers fabricated dual fluorescence electrospun nanofibers using binary blends of poly(2-hydroxyethyl methacrylate-*co*-N-methylolacrylamide-*co*-nitrobenzoxadiazolyl derivative) (poly(HEMA-*co*-NMA-*co*-NBD)) and a spirolactam rhodamine derivative (SRhBOH) [141]. Briefly, the HEMA has been used for hydrophilic properties, and NMA has been used for chemical crosslinking, whereas NBD has been selected for fluorescence-exhibiting characteristics. The free radical copolymerization approach has been used for the synthesis of poly(HEMA-*co*-NMA-*co*-NBD), which has been further blended with SRhBOH and then electrospun to obtain nanofibers that have been further subjected to chemical crosslinking to enhance their stability and use in sensing experiments (see Figure 23). The sensing experiments indicated that upon exposure to Fe^3+^ NBD (FRET donor), emission at 537 nm gradually decreased and SRhBOH (FRET acceptor) emission at 594 nm increased as the concentration increases, owing to the meta ion chelation characteristics. The emission of the nanofibers changed from green to red, which was attributed to the energy transfer from NBD (donor) to SRhBOH (acceptor). The selective response over other ions demonstrated a good selective response toward Fe^3+^, and the fluorescence has been recovered by the treatment of EDTA [141]. Comparable efforts have been also made using multifunctional triblock copolymers, poly(1-pyrenemethylmethacrylate)-*block*-poly(N-isopropylacrylamide)-*block*-poly(N-methylolacrylamide) (PPy-*b*-PNIPAAm-*b*-PNMA) and poly(methyl methacrylate-*co*-rhodamine and quinolone) (poly(MMA-*co*-RQ)) for the detection of Fe^3+^; furthermore, the sensing performance of these nanofiber-based sensors showed improved performances compared with that of the thin film [142,143].

In another study, free radical polymerization has been used to synthesize poly(vinylbenzyl chloride) (PVBC), which has been further dissolved in DMF/THF solvent mixture and further subjected to an electrospinning process; then, the obtained nanofibers have been functionalized with imidazole derivative 2-(2′-pyridyl)imidazole (PIMH) and used in the detection of Fe^2+^ [144]. The functionalized electrospun nanofibers were cut into circular shaped fibers with diameters of 0.9 cm and 1.4 cm, and the corresponding results indicated a significant color change from colorless to red-orange owing to the formation of a low spin six-coordinate complex. The membrane showed excellent selectivity toward Fe^2+^ over a range of mono-, bi-, and trivalent cations tested, and the detection limit was noted as 2 µg mL^−1^ [144]. Another demonstration has been performed by immobilizing carbon quantum dots (CDs) in mesoporous silica/polyacrylonitrile electrospun nanofibrous membrane (CDs/mesoSiO_2_/PAN) and used in the determination of Fe^3+^ [145]. In a brief description, PAN nanofibers have been prepared through electrospinning and further coated with CDs and mesoSiO_2_, which has been further used for the detection of Fe^3+^. The fluorescent behavior of the coated CDs showed enhanced photoluminescence performance because of the possible hydrogen bonding between the SiOH groups in nanofibers and the surface oxygen-containing groups of the CDs, which prevents the defect states of CDs and acts as an active site for the Fe^3+^ adsorption. The sensing experimental results demonstrated that significant fluorescence quench has been noted with a lowest detection limit of 3.95 µM. Further, the fluorescence of the CDs/mesoSiO_2_/PAN nanofibrous membrane has been barely affected by other ions, and the improved selectivity was attributed to the coordinated interaction between Fe^3+^ and the phenol hydroxyl groups of CDs. In order to obtain the insight on their practical utility, detection performances have been also tested using tap water spiked with Fe^3+^ solution [145].

### 2.9. Detection of Other (Silver, Aluminum, and Cesium) Ions

In parallel to the enormous effort made on the electrospun nanofibers-based detection of metal ions described earlier, considerable attempts also have been made on the detection of silver (Ag), aluminum (Al^3+^), and cesium (Cs^+^). As the silver has been used in a large range of products owing to their anti-microbial potential, simultaneously, their increased environmental accumulation has also raised significant concern. In this connection, Kacmaz and co-workers fabricated electrospun nanofibers containing poly(methyl methacrylate) (PMMA) or ethyl cellulose (EC) and methoxy azomethine ionophore (M-AZM) for the detection of Ag^+^ [146]. The sensing experiments demonstrated concentration-dependent fluorescence quenching behavior upon exposure to Ag^+^. The sensing mechanism can be explained by the selective extraction of Ag^+^ into the membrane in the presence of potassium tetrakis-(4-chlorophenyl) borate and further quenches the fluorescence of M-AZM dye with detection limits of 0.46 and 0.34 fmol for the membranes fabricated with EC and PMMA [146]. A similar attempt has also made on the fabrication of colorimetric sensor for the detection of Al^3+^, since it also capable of causing severe impacts including Parkinson’s and Alzheimer’s disease [147]. For this purpose, a rhodamine-based probe (R2PP) has been blended with polyurethane for producing electrospun nanofibers and used in the Al^3+^ detection; the outcome resulted in a detection limit of 8.5 nM [148]. Cesium (Cs^+^) is another important pollutant of the environment that occurs from medical, industrial, and nuclear wastes and contributes to several heath complications including cardiovascular disease [149]. Jung and co-workers used bis(ethylacetate)-bearing calix [4] arene-based chemoprobe embedded PMMA nanofibers for the colorimetric detection of Cs^+^, and the outcome resulted with fluorescence quenching behavior after following exposure to Cs^+^ [150]. Another investigation used 2,3,4-trihydroxybenzaldehyde (BTA) incorporated poly(methylmethacrylate) (PMMA) electrospun nanofibers and employed in the Cs^+^ detection. The UV–vis absorption spectrum of the functional molecule-incorporated nanofibrous membrane showed a maximum at 330 nm; furthermore, it has been shifted to a longer wavelength following the addition of Cs^+^ and turned yellow in color with a detection limit of 44.95 ppm, which was attributed mainly to the cation–π interaction as well as π–π stacking [151].

## 3. Discussion, Conclusions and Future Outlook

Considering all the investigations, progress in the development of electrospun nanofibers-based colorimetric detection of metal ions has mainly adopted direct blending and surface decoration approaches, while very few studies used their combination to functionalize a variety of sensing probes ranging from organic molecules to noble metal nanoclusters. The developed sensing platforms were capable of detecting the metal ions both in qualitative (visual) and quantitative (spectrometric) manner within a few seconds, even at picomolar concentrations, which exemplifies the importance and applicability of the sensors (Table 1, Table 2, Table 3 and Table 4). Most of the sensing probes developed for functionalization purposes hold typical characteristics to interact with specific metal ions using distinctive mechanisms, thus resulting in good sensitivity and selectivity over a variety of competent interferences. It is worth highlighting that the developed sensing platforms were also shown to detect multiple metal ions, which is considered an important factor in designing the metal ion sensors as it could also reduce the cost of sensor fabrication in the detection of different metal ions in polluted water. To this end, the study that functionalized 1,4-dihydroxyanthraquinone into cellulose nanofibers was initially employed in the detection of Cu^2+^ and was further used to detect Cr^3+^ [76]. Likewise, there are many other studies noted in this regard for using the same platforms in the detection of Cu^2+^, Hg^2+^ [86], and Fe^3+^ [82,108,115]. In an in-depth view, appreciable progresses have been made toward detecting most toxic ions including Cu^2+^, Hg^2+^, and Pb^2+^, whereas very limited attempts were directed toward detecting Cr^3+^ and Cd^2+^. Furthermore, among the functionalization approaches used, the surface decoration approach was shown to have improved performances over direct blending, as can be evidenced from the studies performed to detect Hg^2+^ by using the same functional probe [107,117]. The representative studies have been performed using PVA and PCL electrospinning nanofibers functionalized with BSA-capped fluorescent gold nanoclusters through direct blending and surface functionalization approaches, respectively, and the corresponding sensing studies revealed that an enhanced performance has been observed for the nanofibrous sensing platform functionalized with surface decoration with a detection limit of 50 ppt in 10 s. Meanwhile, the direct blending approach was shown to have the lowest detection limit of 1 ppb with a 2-min response time [107,117]. Additionally, on a brief note, the nanofibrous matrix prepared through the polymerization process followed by electrospinning would add an additional complex procedure in the fabrication of sensors while several synthetic polymers available for similar purposes have equal competence. Although appreciable efforts have been made toward fabricating electrospun nanofiber-based sensors for the detection of heavy metal ions at laboratory scale over the decade, no transformations to the real-time applications have been achieved to date.

To conclude, remarkable progress has been made in the design and development of electrospun nanofibers for the colorimetric detection of various metal ions with in the past decade by combining the beneficial features of different functional molecules and electrospun nanofibers that rapidly advanced the field. The present review provides a summary of the cutting-edge efforts progressed on the electrospun nanofibers-based colorimetric detection of various metal ions in water with representative examples, and these promising studies foreshadow the potential applications of electrospun nanofibers in the detection of metal ions that pose severe threats to human and environmental health. Based on the discussions made throughout in this review, it is obvious that the electrospun nanofibers offer excellent features to functionalize a variety of probes through different approaches for the colorimetric detection through the easy visual observation of change in color upon exposure to metal ions, which undeniably showcases the universal nature of colorimetric sensors fabricated using the electrospinning approach. Although several necessary foundation research studies have been established, a substantial body of future research efforts are undoubtedly to be done with a focus on various aspects. According to the extensive evaluation conducted on the literature discussed in the review and based on the author’s expertise in the field, we provide a few directions to explore future research activities to bring the field a step forward. As the colorimetric sensing applications mainly concern enhanced optical characteristics with excellent sensitivity and selectivity, response time, stability, low cost, and reusability, therefore, meticulous efforts are necessary to develop more functional probes addressing the above concerns, and suitable functionalization approaches would also be explored to combine these functional probes with electrospun nanofibers without compromising their original characteristics, which paves an obvious opportunity to employ them in real-world applications. In another aspect, cemented efforts are compulsory for deep understanding on the interaction nature of functional probes/metal ions and the change in colorimetric response induced by metal ions that helps in designing the simultaneous detection of various metal ions, which is very limited in the field, as can be noted from the discussions of the present review and considered as a more important parameter for the purpose of real-time application. Furthermore, concreted approaches need to be laid on the upscaling of the electrospun nanofiber-based sensor that can be produced at an industrial scale. Overall, the future research efforts must be fascinatingly directed toward transforming the research achievements raised out of academic curiosity to real-world applications.

## Figures and Tables

**Table 1:** List of studies progressed for the detection of copper ions using different polymeric matrix and functional molecules approaches along with their performances.

Fibrous Matrix	Functional Molecule	Encapsulation Approach	Limit of Detection (LOD)	Response Time	Ref
Polyamide-6/PANI	PANI leucoemeraldine base	Direct blending	1 ppb	30 min	[71]
Poly(methyl methacrylate-*co*-1,8-naphthalimide)	1,8-naphthalimide	Direct blending	20 × 10^−6^ M	-	[72]
Poly[(N-isopropylacrylamide)-*co*-(N-hydroxymethyl acrylamide)-*co*-(4-rhodamine hydrazonomethyl-3-hydroxy-phenyl methacrylate)]	4-rhodamine hydrazonomethyl-3-hydroxy-phenyl methacrylate	Direct blending	-	-	[73]
Poly(2-hydroxyethyl methacrylate-*co*-N-methylolacrylamide)	9,9-dihexylfluorene-2,7-bipyridine	Direct blending	-	-	[74]
Poly(N-isopropylacrylamide-*co*-N-hydroxymethyl-acrylamide)	1,10-phenanthroline	Direct blending	-	-	[75]
Cellulose	1,4-Dihydroxyanthraquinone	Direct blending	3 nM	-	[76]
Poly(ether sulfones)	Rhodamine dye (spirolactam moiety)	Direct blending	1.1 × 10^−9^ M	10 min	[77]
Cellulose Acetate	Rhodamine B derivative	Direct blending	18 ppm	<100 s	[78]
Poly(methyl methacrylate) (PMMA) Ethyl cellulose (EC)	N’-3-(4-(dimethylamino phenly)allylidene) isonicotinohydrazide	Direct blending	3.8 × 10^−14^ M for EC 1.4 × 10^−13^ M for PMAA	-	[79]
Polyvinyl alcohol (PVA)/tetraethyl orthosilicate (TEOS)	Schiff base	Direct blending	1.27 × 10^−8^ mol L^−1^	90 s	[80]
Polysuccinimide	Poly (aspartic acid)	Direct blending	0.01 mg/L	5 min	[81]
Polysuccinimide	Poly (aspartic acid)	Direct blending	0.3 mg/L	-	[82]
Polymethyl methacrylate	CsPbBr_3_ perovskite quantum dots and cyclam	Direct blending and surface functionalization	10^−15^ M	-	[83]
Poly(vinylidene fluoride)	Quantum dots modified with polyethylenimine	Direct blending and surface functionalization	2 µM	30 s	[84]
Poly (MMA-*co*-AHPA)	Rhodamine B-hydrazine	Surface functionalization	1.5 × 10^−6^ mol L^−1^	<10 s	[85]
Poly(HEMA-*co*-NMA	2-((pyren-1-yl)methyleneamino)-3-amino maleonitrile) (PyDAN2)	Surface functionalization	10^−7^–10^−6^ M	-	[86]
Ethylene-vinyl alcohol copolymer (EVOH)	4-aminobenzoic acid (PABA) and 1-pyrenecarboxaldehyde (Py-CHO)	Surface functionalization	1 × 10^−9^ M	-	[87]
Cellulose	1,4-Dihydroxyanthraquinone and carbon nanotubes	Direct blending and Surface functionalization	2.17 × 10^−9^ M	6 min	[88]
Polyacrylonitrile	Gold/Silver core/shell nanoparticles	Surface functionalization	50 nM	3 min	[89]
Cellulose acetate	Dithiothreitol capped gold nanocluster	Surface decoration	50 ppb	10 min	[90]
Polyacrylonitrile	Nitrogen-doped carbon dots	Carbonization	5 nM	-	[91]

“-” denotes the specific parameter has not been reported/found.

**Table 2:** List of studies progressed for the detection of lead ions using different polymeric matrix and functional molecules approaches along with their performances.

Fibrous Matrix	Functional Molecule	Encapsulation Approach	Limit of Detection (LOD)	Response Time	Ref
Polyacrylonitrile	Polydiacetylene	Direct blending	0.48 µM	30 min	[92]
Polyacrylonitrile	Polydiacetylene	Direct blending	0.24 mM	10 min	[93]
Cellulose acetate	Curcumin	Direct blending	0.12 ± 0.01 µM	-	[94]
Cellulose acetate	Curcumin nanoparticles	Direct blending	0.14 ± 0.01 mM	-	[95]
Polyamide-6 /Nitrocellulose	Bovine serum albumin decorated gold nanoparticles	Surface functionalization	0.2 µM	60 min	[96]
Nylon-6/Polyvinylidene fluoride	Glutathione conjugated gold nanoparticles	Surface functionalization	10 µg/dL	10 min	[97]
Deacetylated cellulose acetate	Pyromellitic dianhydride	Surface functionalization	0.048 µM	-	[98]
Polyacrylonitrile	Silver and silica	Direct blending/ Surface functionalization	-	-	[99]

“-” denotes the specific parameter has not been reported/found.

**Table 3:** List of studies progressed for the detection of mercury ions using different polymeric matrix and functional molecules approaches along with their performances.

Fibrous Matrix	Functional Molecule	Encapsulation Approach	Limit of Detection (LOD)	Response Time	Ref
Polyvinyl alcohol	Bovine serum albumin (BSA)-capped fluorescent gold nanoclusters (AuNC)	Direct blending	1 ppb	2 min	[107]
Poly(acrylic acid)−poly(pyrene methanol) (PAA−PM)	Poly(pyrene methanol)	Direct blending	-	-	[108]
Polyacrylonitrile	Dithioacetal-modified perylenediimide (DTPDI)	Surface functionalization	1 ppb	2 h	[109]
Polyvinyl alcohol - tetraethyl orthosilicate	Carbazol-based Schiff base (S)	Direct blending	0.018 ppb	60 s	[110]
Poly(2-hydroxyethyl methacrylate-*co*-N-methylolacrylamide-*co*-rhodamine derivative)	Rhodamine derivative	Direct blending	10^−7^ M	2 min	[111]
Poly(N-isopropylacrylamide)-*co*-(N-methylolacrylamide)-*co*-(Acrylic acid)	1-benzoyl-3-[2-(2-allyl-1,3-dioxo-2,3-dihydro- 1Hbenzo[de] isoquinolin-6-ylamino)-ethyl]-thiourea	Direct blending	10^−3^ M	-	[112]
Gold nanoparticles, rhodamine B, and TEOS	Gold nanoparticles and rhodamine B (RhB)	Direct blending	1.10 nM	-	[113]
Poly(HEMA-co-NMA)	Pyrene derivative (PyDAN2) or rhodamine B derivative (RhBN2)	Surface functionalization	10^−2^–10^−1^ M	-	[86]
(poly(methyl methacrylatete-*co*-1,8-naphthalimide derivatives-*co*-rhodamine derivative)	Rhodamine derivative	Direct blending	2 × 10^−8^ M	-	[114]
Polymethyl methacrylate	Hexaphenylsilole	Direct blending	-	-	[115]
Ethyl cellulose	4-(dimethylamino)benzaldehyde 2- [[4-cyanophenyl] methylene]hydrazone dye (DC-AZM)	Direct blending	0.07 nM	-	[116]
Polycaprolactone	BSA-capped fluorescent gold nanoclusters (AuNC)	Surface functionalization	50 ppt	10 s	[117]
Polymer blends of hydroxyl monomers	Spirocyclic Rhodamine 6G phenyl-thiosemicarbazide derivative	Direct blending	0.1 µM	15 min	[118]
Ethyl cellulose	2-(9-methyl-9H-carbazol-3-yl)-5-(pyridin-4-yl)- 1,3,4-oxadiazole (ODC-3) dye	Direct blending	1.70 × 10^−15^ M	-	[119]
Polyacrylonitrile	Silver and Silica	Direct blending/ Surface decoration	-	-	[99]
Polyaniline	Polyaniline leucoemeraldine base (PANI-LB)	Direct blending	5 nM	20 min	[120]
Poly (MMA-co-ADMA)	Rhodamine–β-cyclodextrin	Surface functionalization	6.0 × 10^−5^ mol L^−1^	<1 min	[121]

“-” denotes the specific parameter has not been reported/found.

**Table 4:** List of studies progressed for the detection of iron ions using different polymeric matrix and functional molecules approaches along with their performances.

Fibrous Matrix	Functional Molecule	Encapsulation Approach	Limit of Detection (LOD)	Response Time	Ref
Poly(acrylic acid)	Poly(pyrene methanol)	Direct blending	1.1 × 10^6^ M^−1^	-	[108]
Polymethyl methacrylate	Hexaphenylsilole (HPS)	Direct blending	-	-	[115]
Zein	Curcumin	Direct blending	0.4 mg/L	3 h	[135]
Zein	Curcumin	Direct blending	0.3 mg/L	0.5 h	[136]
Ethyl cellulose	N’-(4-cyanobenzylidene) isonicotinohydrazide (CBINH)	Direct blending	0.07 pM	<30 s	[137]
Polycaprolactam	1,10-phenanthroline	Direct blending	1 µg/mL	-	[138]
Polycaprolactone	4,4′-Fluoresceinoxy bisphthalonitrile	Direct blending	2.9413 nM	1 min	[139]
Polysuccinimide	Poly (aspartic acid)	Direct blending	0.1 mg/L	-	[82]
Polyacrylonitrile	Silver and silica	Direct blending/ Surface functionalization	-	-	[99]
Poly(methyl methacrylate) and poly(vinyl chloride-*co*-vinyl acetate-*co*-vinyl alcohol)	Pyrene	Direct blending	-	-	[140]
Poly(2-hydroxyethyl methacrylate-*co*-N-methylolacrylamide-*co*-nitrobenzoxadiazolyl derivative)	Spirolactam rhodamine derivative	Direct blending	10^−4^ M	15 min	[141]
Poly (methyl methacrylate-co- rhodamine and quinolone)	Poly(1-pyrenemethylmethacrylate)	Direct blending	2 × 10^−5^ M	-	[142]
Poly(methyl methacrylate-*co*- rhodamine and quinolone)	Rhodamine	Direct blending	1.19 µM	<1 min	[143]
Poly(vinylbenzyl chloride)	2-(2′-pyridyl)imidazole	Surface functionalization	2 µg mL^−1^	-	[144]
Polyacrylonitrile	Carbon quantum dots	Surface functionalization	3.95 µM	-	[145]

“-” denotes the specific parameter has not been reported/found.
